# Hot‐Pressing Annealing‐Induced Light Utilization Enhancement and Crystallinity Optimization Enable High‐Performance Narrowband Ultraviolet Photodetectors for Real‐Time Ultraviolet Radiation Monitors

**DOI:** 10.1002/advs.202513795

**Published:** 2025-10-06

**Authors:** Jingli Ma, Junhao Zhu, Conghui Dun, Hao Wang, Bangbang Yang, Mengyao Zhang, Lejin Li, Huifang Ji, Yanbing Han, Ying Liu, Di Wu, Xinjian Li, Chongxin Shan, Zhifeng Shi

**Affiliations:** ^1^ Key Laboratory of Materials Physics of Ministry of Education School of Physics Zhengzhou University Daxue Road 75 Zhengzhou 450052 China

**Keywords:** Cs_3_Cu_2_I_5_, hot‐pressing, narrowband ultraviolet photodetector, optical field distributions, ultraviolet radiation monitor

## Abstract

The real‐time ultraviolet (UV) radiation monitor can protect personal healthcare by real‐time monitoring UV radiation, highlighting the urgent need for high‐performance self‐powered narrowband UV detectors that can selectively response to UVA and UVB wavelengths. Existing narrowband photodetectors often rely on a surface defect‐assisted charge collection narrowing strategy, which inevitably compromises device performance. In this study, a hot‐pressing (HP) annealing strategy is introduced to prepare high‐quality Cs_3_Cu_2_I_5_ films with large grains vertically spanning the entire thickness. The HP annealing reduces the defect density and light loss in the 280−375 nm range in the Cs_3_Cu_2_I_5_ films, and enhances the charge transport and collection in the Cs_3_Cu_2_I_5_/GaN heterojunction. As a result, the Cs_3_Cu_2_I_5_/GaN‐based photodetectors achieve self‐powered UV detection performance with low dark current (1.50 × 10^−12^ A), large responsivity (0.22 A W^−1^), high detectivity (6.40 × 10^11^ Jones), and fast response speed (26/117 µs), while maintaining narrowband detection capabilities (280−375 nm). Furthermore, the devices implement a real‐time and continuous UV radiation monitor to help prevent diseases caused by excessive UV radiation. This work not only propels the evolution of high‐performance narrowband UV photodetectors but also links technological innovation with equipment designed for practical applications.

## Graphical abstract

By utilizing a hot‐pressing (HP) annealing strategy, Cs_3_Cu_2_I_5_ films with larger grains vertically spanning the entire thickness are fabricated. Because HP annealing enhances light utilization and charge transport in Cs_3_Cu_2_I_5_/GaN heterojunction, the HP device achieves superior detection performance while maintaining narrowband photoresponse to UVA/UVB lights (280–375 nm). Finally, the HP device is successfully applied in real‐time UV radiation monitors.

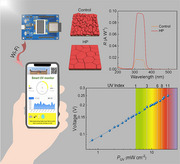

## 1 Introduction

According to statistics from the World Health Organization, solar UV radiation is responsible for over 1.5 million cases of skin cancer annually. Additionally, up to 10% of cataracts may result from excessive UV exposure. UV radiation also suppresses immune responses, for instance, potentially triggering the recurrence of cold sores when the body's immune response to the herpes simplex virus becomes compromised.^[^ ^1^, ^2^, ^3^ ^]^ The sun is the most significant source of environmental UV radiation, with its spectrum divided into three regions: UVA (315−400 nm), UVB (280−315 nm), and UVC (100−280 nm). When sunlight passes through the atmosphere, all UVC is absorbed by ozone, water vapor, oxygen, and carbon dioxide, and does not reach the earth's surface. Only part of the UVB and UVA radiation penetrates the atmosphere to reach the ground.^[^ ^1^ ^]^ Consequently, the UV index, a standard metric for quantifying the potential health risks of solar UV radiation to humans, primarily focuses on UVB and UVA wavelengths.^[^ ^2^, ^3^, ^4^ ^]^ Moderate UVA and UVB exposure is essential for health, as it promotes vitamin D synthesis, helps kill bacteria, and prevents or treats rickets.^[^ ^4^ ^]^ However, excessive exposure to UVA and UVB radiation will cause severe damage to human skin, eyes, and immune system, with heightened risks, particularly for high‐risk groups like children, adolescents, fair‐skinned individuals, and outdoor workers who experience prolonged sun exposure.^[^ ^2^, ^5^, ^6^ ^]^ It is important to note that UV radiation levels are influenced by multiple factors such as solar altitude angle, latitude, cloud cover, ozone concentration, and climate change, resulting in significant regional variations. Therefore, developing real‐time, portable UV radiation monitors is crucial for preventing diseases associated with UV exposure.

The UV radiation monitors hold significant potential for widespread application. In the consumer electronics sector, they can be integrated into smartphones and smartwatches to provide users with real‐time outdoor UV monitoring and protection recommendations. In the health and beauty industry, they can contribute to the development of personalized skincare regimens, helping prevent photo‐aging and reduce skin cancer risks. Furthermore, in the fields of smart home and automotive systems, they can be linked with smart curtains and window shading systems to automatically adjust shading based on UV index (UVI), thereby improving health protection levels. However, currently available high‐precision UV monitors generally suffer from complex structures, large size, and high cost, making them unsuitable for portable applications. On the other hand, most portable UV detectors exhibit limited detection capabilities and poor anti‐interference performance. It is important to note that health risks arise not only from high‐intensity UV exposure but also from the long‐term accumulation of low‐intensity radiation, such as during early morning, late afternoon, or under cloudy conditions.^[^ ^4^ ^]^ The application scenario of simple outdoor detection is not limited to personal daily protection but can also be extended to areas such as protection for high‐risk groups. For instance, in playgrounds or kindergartens, continuous monitoring of all‐day UV exposure is essential to inform the design of appropriate outdoor activity schedules. Insufficient detectivity may lead to significant underestimation of UV exposure levels, resulting in inadequate protective measures. Therefore, high specific detectivity (*D* ^*^) remains critically important. In addition, although UV radiation monitors are not affected by natural UVC interference, the growing prevalence of artificial UVC sources in various settings, such as water treatment facilities, medical environments, and public spaces for air purification, poses a new challenge.^[^ ^7^ ^]^ The artificial UVC light can be scattered and inadvertently detected by the UV sensors. If the UV sensors are response to UVC light, the UV exposure levels and UVI will be overestimated. Therefore, there is a critical need to develop a portable, real‐time UV radiation monitor with selective narrowband detection capability for both UVA and UVB spectra, high specific detectivity, and all‐weather operational capability.

To achieve an interference‐resistant UV radiation monitoring sensor, it is essential to fabricate narrowband UV photodetectors that respond only to specific wavelengths of UVB and UVA. The current strategy for realizing narrowband detection primarily is to combine broadband detectors with dichroic prisms or optical filters, which will increase the costs, complexity, and physical volume of the detection system. Besides, narrowband detection can be achieved by unitizing surface plasmon resonance effects through the deposition of quantum dots or metal particles, such as Al or Mg, but the susceptibility of these metals to oxidation can deteriorate sensitivity and resolution.^[^ ^8^, ^9^ ^]^ The charge collection narrowing (CCN) strategy has garnered significant attention for its ability to achieve narrowband detection by leveraging the intrinsic properties of materials.^[^ ^10^, ^11^, ^12^, ^13^, ^14^ ^]^ For instance, Xu et al. demonstrated chalcogenides‐based narrowband photodetectors through a filterless CCN strategy and fabricated a set of red, green, blue, and near infrared narrowband photodiodes.^[^ ^10^ ^]^ However, due to the bandgap limitations of the materials, the response spectrum of the detectors is not suitable for use in UV radiation monitors.

Metal halide perovskites, with simple manufaction processes, tunable bandgap, and outstanding optoelectronic properties, are highly suitable for narrowband photodetectors.^[^ ^11^, ^14^, ^15^ ^]^ For instance, Wang et al. developed a self‐powered narrowband photodetector with tunable spectral responses based on MAPbI_x_Br_3−x_ films utilizing the CCN strategy.^[^ ^16^ ^]^ However, traditional perovskites have limitations in narrowband UV detection applications due to lead toxicity, instability, and an inherent narrow bandgap. In this context, lead‐free inorganic perovskite derivatives Cs_3_Cu_2_I_5_ offer unique advantages for narrowband UV detection, including a big direct bandgap of 3.8 eV, high light absorption, long carrier lifetime, and excellent stability in ambient air.^[^ ^17^, ^18^, ^19^, ^20^ ^]^ Li et al. achieved a filterless narrowband UV photodetector based on spin‐coated Cs_3_Cu_2_I_5_ thick films utilizing the CCN strategy.^[^ ^21^ ^]^ However, conventional spin‐coating technique often results in numerous grain boundaries, pores, and disorderly grain orientation in Cs_3_Cu_2_I_5_ films due to the rapid nucleation and crystallization process, which inevitably produces superfluous defect states. Although the CCN strategy relies on the recombination of surface defect states, the excessive number of defect states will undermine the efficiency and stability of the photodetectors.^[^ ^22^ ^]^ Therefore, reducing defect state density in Cs_3_Cu_2_I_5_ films is crucial to achieving an optimal balance between narrowband detection capability and overall device performance. The pressure is one of the important parameters of the thermodynamic and kinetic properties, and has been pointed out as an effective approach to access the structure‐property relationships of perovskites,^[^ ^23^, ^24^ ^]^ which can affect their morphology, crystallization, and optoelectronic performances.^[^ ^25^, ^26^, ^27^, ^28^, ^29^ ^]^

In this study, a hot‐pressing annealing strategy was employed to achieve high‐quality Cs_3_Cu_2_I_5_ films (referred to as HP films later) with few grain boundaries, high crystallinity, and vertically aligned grains. By integrating the HP films with GaN, high‐performance narrowband (280−375 nm) photodetection capability was demonstrated. Notably, the suppression of photon scattering in the 280−375 nm range within the HP films enhances photon penetration near the Cs_3_Cu_2_I_5_/GaN interface, thereby promoting the generation of photo‐induced carriers. The HP films also establish a higher built‐in electric field with GaN, enabling more efficient separation and transport of photogenerated carriers. Besides, the superior carrier transport properties along the vertically aligned grains in HP films provide more a chance for effective collection of photogenerated holes. Collectively, the synergistic interaction of these factors significantly enhances the narrowband UV photodetection performances. Consequently, the photodetector based on HP films/GaN heterojunction achieves a lower dark current (1.50 × 10^−12^ A), a higher responsivity (0.22 A W^−1^), a larger specific detectivity (6.40 × 10^11^ Jones), and a faster response speed (26/117 µs). Importantly, the photodetector culminates in the successful implementation of a real‐time UV radiation monitor.

## 2 Result and Discussion

**Figure 1:**
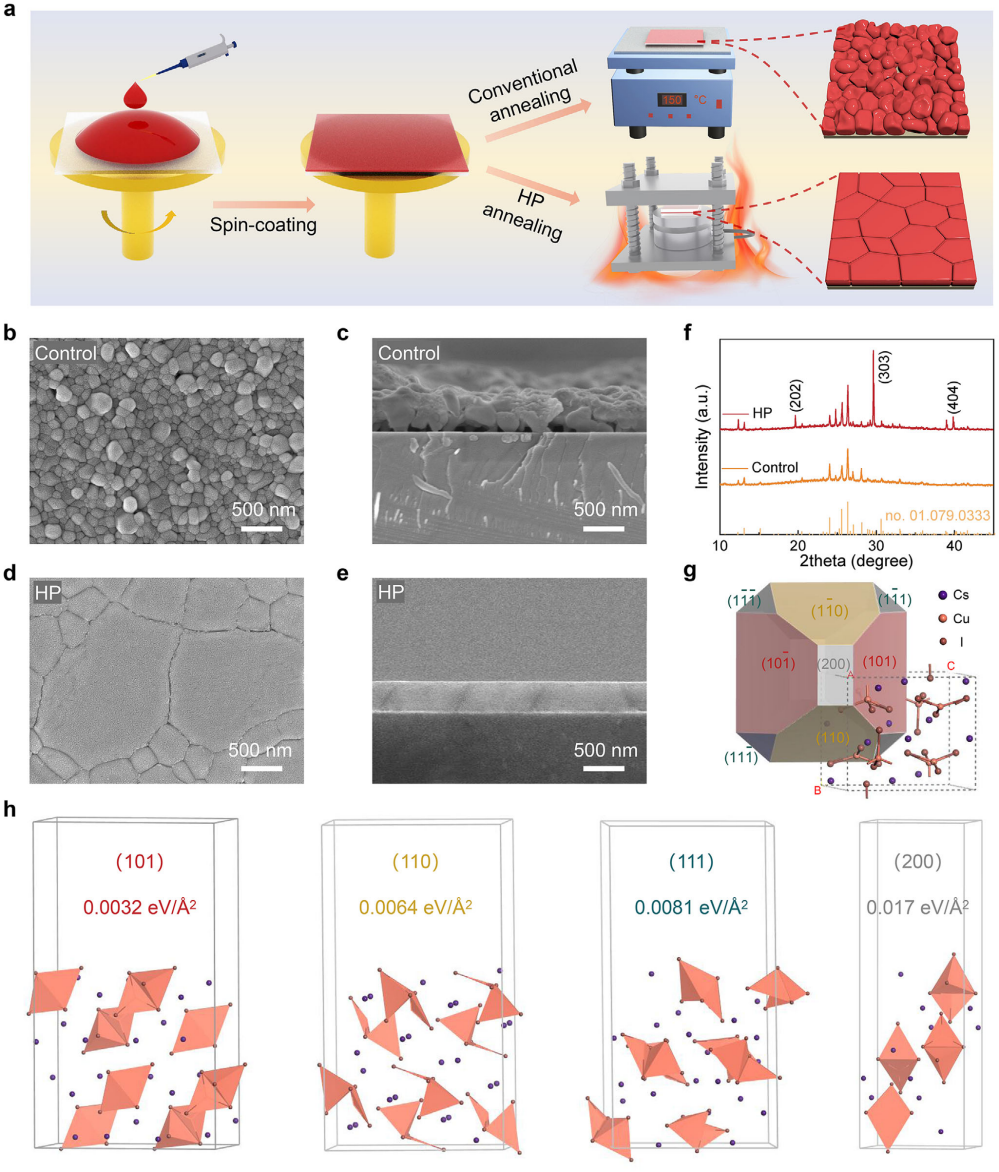
a) Schematic diagram of the preparation of Cs_3_Cu_2_I_5_ films through conventional and HP annealing methods. Top‐view SEM images of b) control films and c) HP films. Cross‐sectional SEM images of d) control films and e) HP films. f) XRD patterns and g) predicted morphology of Cs_3_Cu_2_I_5_ single crystal using the BFDH method. h) Surface energy of (101), (110), (111), and (200) crystal faces of Cs_3_Cu_2_I_5_.

**Figure** **1**a illustrates the processes of conventional annealing and HP annealing for preparing Cs_3_Cu_2_I_5_ films. First, Cs_3_Cu_2_I_5_ precursor films were prepared by a spin‐coating process. Then they were placed on a heating plate at 150 °C and annealed for 1 h (conventional annealing). For comparison, a counterpart sample was covered with a silicon wafer and subjected to a pressure of 180 MPa at 150 °C for 1 h (HP annealing). Compared with the control films prepared by conventional annealing, the HP films reveal bigger grain size, fewer grain boundaries, and negligible holes, as seen in Figure 1b,d. Moreover, the HP films, with larger lateral dimension grains, bind more tightly to the underlying GaN (Figure 1c,e), which favors reducing interfacial resistance and enhancing the transmission of photogenerated carriers. Figure 1f presents the X‐ray diffraction (XRD) patterns of control films and HP films. One can see that the HP films emerge three diffraction peaks at 19.57°, 29.54°, and 39.74°, corresponding to the (202), (303), and (404) planes of orthorhombic Cs_3_Cu_2_I_5_, respectively, demonstrating preferred growth along the (101) crystal plane. To study the growth mechanisms, we predicted the morphology of Cs_3_Cu_2_I_5_ single crystal using the Bravais–Friedel and Donnay–Harker (BFDH) methods. As presented in Figure 1g and Table S1 (Supporting Information), the predicted morphology consists of the (101), (110), (111), and (200) crystal planes, with the (101) crystal plane occupying the largest portion of the total crystal planes area. Further, we calculated the surface energy of these crystal planes. As presented in Figure 1h, the (101) crystal plane presents the lowest surface energy of 0.0032 eV Å^−2^. According to the Wulff's theorem, the external surface of the crystal follows the principle of minimum surface energy, and the final external morphology is dictated by the number of crystal faces with low surface energy and their growth rates.^[^ ^30^, ^31^, ^32^ ^]^ Compared with other crystal planes, the (101) crystal plane with the lowest surface energy has the slowest growth rate due to its lower tendency to adsorb atoms, resulting in the (101) crystal plane dominating the final morphology. This is consistent with the emergence of preferred growth of the (101) crystal plane for HP films observed in XRD, which is strong evidence of the rearrangement, fusion, and recrystallization of grains in the HP annealing process.

From a crystal growth perspective, the HP method enhances the crystallographic orientation and crystallinity of Cs_3_Cu_2_I_5_ films by applying additional pressure that modifies growth thermodynamics and kinetics, in contrast to conventional annealing, which relies solely on thermal energy. Thermodynamically, the applied pressure enhances the chemical potential difference between small and large grains.^[^ ^33^ ^]^ This increased energy differential accelerates Ostwald ripening, favoring the dissolution of smaller, high‐energy grains and the preferential growth of larger, more stable ones. Moreover, within the spatially confined environment, grains with the (101) plane, identified as the lowest surface energy plane, orient themselves parallel to the substrate to minimize the system surface energy, thereby establishing the thermodynamic basis for preferred orientation.^[^ ^34^ ^]^ Kinetically, the pressure facilitates the migration of solute atoms and ions along the pressure direction, facilitating the formation of vertically aligned grains that span the entire film.^[^ ^35^ ^]^ Concurrently, the mechanical compression rapidly eliminates pores formed during solvent evaporation, achieving rapid densification and providing a favorable medium for subsequent grain growth. The geometric constraint imposed by the confined space suppresses out‐of‐plane growth and encourages in‐plane grain competition. Grains with preferred orientations gradually consume misoriented neighbors, leading to the development of a highly oriented, large‐grained morphology.^[^ ^35^ ^]^ In conclusion, HP annealing enables the preparation of high‐quality Cs_3_Cu_2_I_5_ films characterized by excellent densification, substantially enlarged grain size, and a strong (101) preferred crystallographic orientation. The synergistic coupling of thermal and mechanical energy provided by HP annealing may be a general approach for enhancing the crystallinity of 0D metal halide films.^[^ ^36^ ^]^

We measured the steady‐state photoluminescence (PL) and PL excitation (PLE) spectra of both the HP films and control films. As shown in Figure s1 (Supporting Information), both exhibit a broad PL peak at 445 nm and a large Stokes shift, which indicates that the emission feature of Cs_3_Cu_2_I_5_ can be explained via exciton self‐trapping.^[^ ^18^ ^]^ Figure S2 (Supporting Information) illustrates the possible recombination kinetics. Upon photo‐excitation, electrons quickly relax to the conduction band edge, forming free carriers. Due to the strong photo‐acoustic coupling, the lower‐energy self‐trapping exciton (STE) state will capture free electrons. Radiative recombination occurs as electrons transition from the STE state to the ground state, resulting in blue emission with a large Stokes shift. Besides, some free electrons may be captured by trap states, leading to non‐radiative recombination. In addition, the HP films show an enhanced PL intensity, indicating that the HP process effectively reduces nonradiative recombination channels and promotes radiative recombination of STEs.^[^ ^37^, ^38^ ^]^

To evaluate the recombination kinetics of photogenerated carriers, we therefore measured the time‐resolved PL spectra of the control and HP films. As shown in Figure S3 (Supporting Information), two curves can be fitted by a biexponential function, with detailed fitting parameters summarized in Table S2 (Supporting Information). The fast component (*τ*_1_) is associated with the trap‐assisted nonradiative recombination near the grain boundaries, while the slow component (*τ*_2_) corresponds to the radiative recombination from bulk Cs_3_Cu_2_I_5_. Compared with the control films, the HP films exhibit a longer weighted‐average lifetime (*τ*_ave_.), suggesting the HP annealing can reduce the surface defect states and nonradiative recombination centers, favoring an efficient utilization of photogenerated carriers. To quantificationally evaluate the ion migration possibility in Cs_3_Cu_2_I_5_ films, we further analyzed the relationship between dark current (*I*_D_) and temperature to get the activation energy of ion migration (*E*_a_). Figure S4 (Supporting Information) plots the data fitted using the Arrhenius equation of *I*_D_ = *I_D_*_0_∙exp(−*E*_a_/*k*_B_ *T*), where *I_D_*_0_, *k*_B_, and *T* are the proportional constant, Boltzmann constant, and temperature, respectively. In contrast to the control films, the HP films exhibit a relatively larger *E*_a_, suggesting the suppressed ion migration that favored an enhanced device stability. To quantify the semiconductor performance of Cs_3_Cu_2_I_5_ films, the current–voltage (*I*−*V*) curves of hole‐only devices were carried out in the space‐charge‐limited current (SCLC) mode. As illustrated in Figure S5 (Supporting Information), the curves (*I* ∝ *V^n^*) can be divided into three regions: Ohmic region (*n* = 1), trap‐filled limit voltage region (*n* > 2), and Child region (*n* = 2). The trap state density (*n*_trap_) can be determined using the formula of *n*_trap_ = 2*ε*_0_ *ε*_r_ *V*_TFL_/*qL* ^2^, where *ε*_0_, *ε*_r_, *V*_TFL_, *q*, and *L* are the vacuum dielectric constant, relative dielectric constant, trap‐filled limit voltage, elementary electronic charge, and the channel width, respectively. Compared to the control films (6.68 × 10^12^ cm^−3^), the HP films exhibit a lower *n*_trap_ of 5.91 × 10^12^ cm^−3^, indicating a better crystal quality. Moreover, the carrier mobility (*µ*) is determined using the Mott–Gurney law of *J*_D_ = 9*ε*_0_ *ε*_r_ *µV* ^2^/8*L* ^3^, where *J*_D_ and *V* are the current density and applied voltage, respectively. The *µ* of the HP films is calculated as 0.07 cm^2^ V^−1^ s^−1^, which is higher than that of the control films (0.04 cm^2^ V^−1^ s^−1^), suggesting that the HP films have better carrier transport capacity.

**Figure 2:**
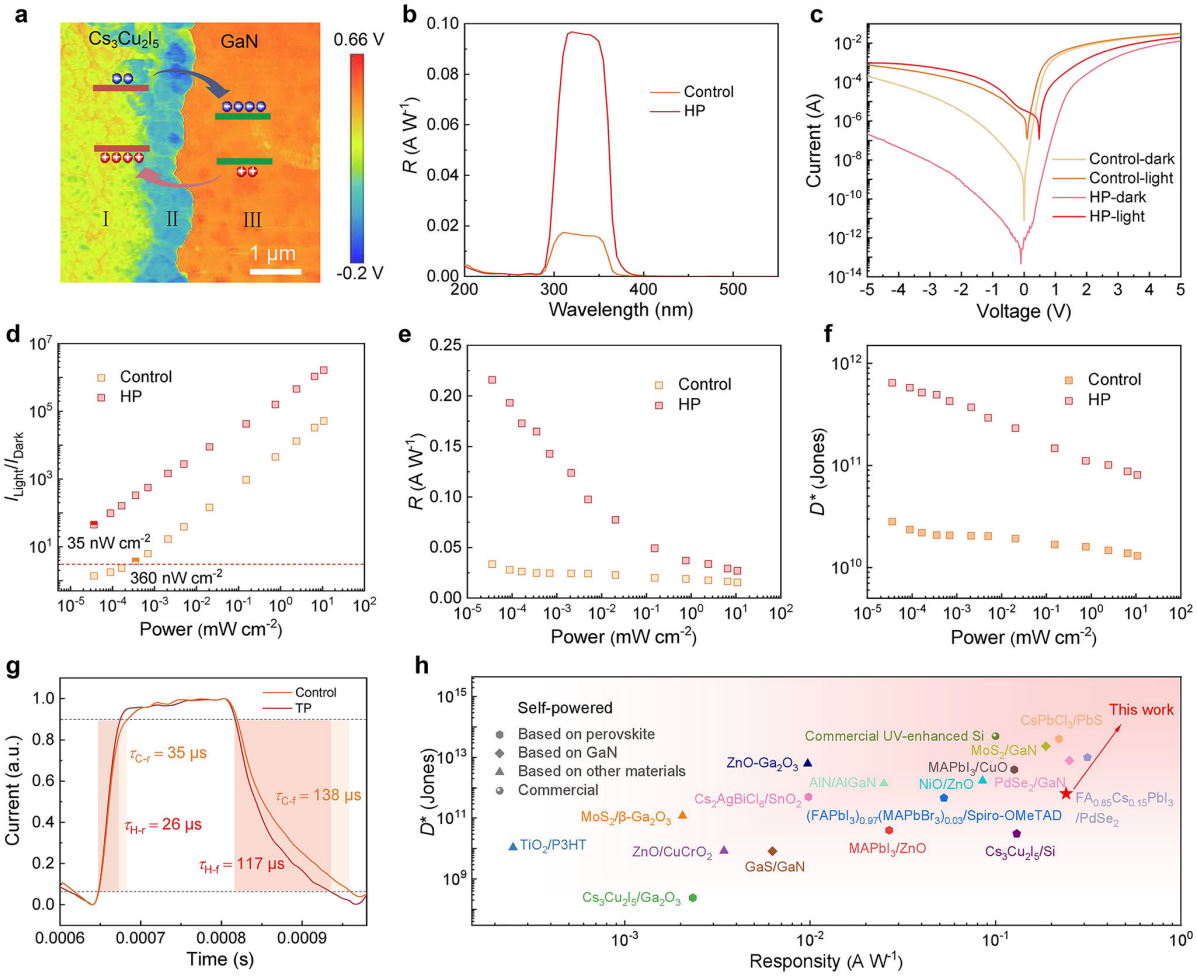
a) Energy band alignment and CPD distribution of the Cs_3_Cu_2_I_5_/GaN heterojunction. b) Response spectra of the control device and HP device. c) *I*–*V* curves of the control device and HP device under dark and 360 nm light excitation with a light power of 12 mW cm^−2^. d) *I*_Light_/*I*_Dark_, e) responsivity, and f) specific detectivity versus light power of the control device and HP device. g) Rise time and fall time of the control device and HP device. h) Comparison of the responsivity and specific detectivity of the HP device with other reported self‐powered UV photodetectors.

As illustrated in **Figure** **2**a, Cs_3_Cu_2_I_5_ and GaN form the type‐II energy band alignment. Cs_3_Cu_2_I_5_ and GaN possess p‐ and n‐type conductivity properties, respectively; upon contact, electrons diffuse from GaN to Cs_3_Cu_2_I_5_, while holes diffuse from Cs_3_Cu_2_I_5_ to GaN, forming a built‐in electric field directed from GaN to Cs_3_Cu_2_I_5_ at the heterointerface. Therefore, the potential in GaN is higher than that in Cs_3_Cu_2_I_5_. Note that this intrinsic field allows the device to operate without an external power supply. The Kelvin probe force microscopy (KPFM) enables nanoscale imaging of surface potential difference (CPD) and charge distribution.^[^ ^39^, ^40^, ^41^ ^]^ To investigate the CPD distribution between Cs_3_Cu_2_I_5_ and GaN, a KPFM test was performed on the Cs_3_Cu_2_I_5_/GaN heterojunction. As shown in Figure 2a and Figure S6 (Supporting Information), the CPD can be divided into three distinct regions. The CPD in region III (pure GaN) is the highest, followed by region I (pure Cs_3_Cu_2_I_5_), and region II (Cs_3_Cu_2_I_5_ on GaN) is the lowest. This aligns with the aforementioned analysis, confirming the existence of a built‐in electric field.

Further, a heterostructured photodetector based on Au/HP films/GaN/In (referred to as the HP device) was constructed. As a comparison, the control device with the structure of Au/control films/GaN/In was also fabricated with identical conditions. Figure 2b shows the response spectra of two devices. One can see that HP annealing not only improves the responsivity of the device, but also maintains the narrowband UV detection characteristic within the 280−375 nm range. Therefore, we selected a 360 nm incident light source to perform a series of photoelectric tests. Figure 2c presents the *I*−*V* curves of the control device and HP device. By contrast, the HP device exhibits a lower dark current, a higher photocurrent, and a bigger rectification ratio. These improvements may be attributed to the reduced leakage current and enhanced collection of photogenerated carriers in the HP device. Figure S7 (Supporting Information) shows the current‐time (*I*−*t*) curves of the control and HP devices at zero bias. Because HP annealing effectively reduces the grain boundaries and defect states, so the dark current of the HP device (1.50 × 10^−12^ A) is nearly an order of magnitude lower than the control device (1.80 × 10^−11^ A). At all comparable light excitation powers, the HP device exhibits higher *I*_Light_/*I*_Dark_ vaules compared to the control device, as seen in Figure 2d, with the *I*_Light_/*I*_Dark_ reaching up to 3.0 × 10^6^ at a light power of 12 mW cm^−2^. Notably, the minimum detection limit (*I*_Light_/*I*_Dark_ = 3) of the HP device is 36 nW cm^−2^, reduced by 10% in comparison with the control device (360 nW cm^−2^).

To assess the detection performance quantitatively, we calculated the responsivity (*R*) of two devices using the formula of *R* = *I*_P_/(*S*·*P*) = (*I*_L_−*I*_D_)/(*S*·*P*), where *I*_L_, *I*_D_, and *S* are photocurrent, dark current, and effective area of the devices, respectively. As presented in Figure 2e, when the incident light power is 0.08 µW cm^−2^, the HP device achieves a maximum *R* of 0.22 A W^−1^, which is 6‐fold higher than that of the control device. The specific detectivity (*D* ^*^), another key parameter for the photodetectors, was calculated using the formulas of $D^{*}=\frac{\sqrt{A\Delta f}}{NEP}$ and $NEP=\frac{\sqrt{\bar{{i_{n}}^{2}}}}{R}$, where *A* is the effective area of the device, ∆*f* is the electrical bandwidth, NEP is noise equivalent power, *R* is responsibility, and $\sqrt{\bar{{i_{n}}^{2}}}$ is the root‐mean‐square value of the noise current.^[^ ^42^ ^]^ Figure S8 (Supporting Information) illustrates the relationship between $\sqrt{\bar{{i_{n}}^{2}}}$ of the control and HP devices in different frequencies, which were acquired from a rapid Fourier transform of the time‐domain dark current. The noise level per unit bandwidth of the control and HP devices was determined to be 1.03 × 10^−13^ and 2.95 × 10^−14^ A Hz^−1/2^, respectively. As presented in Figure 2f, the *D* ^*^ of the HP device was calculated as 6.40 × 10^11^ Jones (1 Jones = 1 cm Hz^1/2^ W^−1^) at a light power of 0.08 µW cm^−2^, which is nearly an order of magnitude higher than that of the control device, showing an exceptional sensitivity of the weak light signals. The external quantum efficiency (EQE), one of the important metrics of the photodetectors, can reflect the conversion efficiency between the input optical signal and the output electrical signal, which can be determined using the following formula of EQE = *Rhv*/*q*, where *h* and *ν* is the Planck constant and the frequency of the incident photons, respectively. As presented in Figure S9 (Supporting Information), the EQE of the control and HP devices were calculated to be 12% and 74%, respectively, demonstrating that the HP device possesses better photoelectric conversion efficiency. According to the photocurrent response profile shown in Figure 2g, the HP device exhibits a faster response (26/117 µs) to optical signals compared to the control device, which can be attributed to the improvement of crystallization and the reduction of defect‐induced nonradiative recombination in the HP films. Figure 2h and Table S3 (Supporting Information) summarize the key performance metrics of the as‐prepared photodetectors with other reported self‐powered UV photodetectors. It can be observed that the HP device exhibits outstanding performances in *R*, *D* ^*^, EQE, and response speed compared to existing counterparts.^[^ ^18^, ^43^, ^44^, ^45^, ^46^, ^47^, ^48^, ^49^, ^50^, ^51^, ^52^, ^53^, ^54^, ^55^, ^56^, ^57^, ^58^, ^59^, ^60^, ^61^, ^62^ ^]^

**Figure 3:**
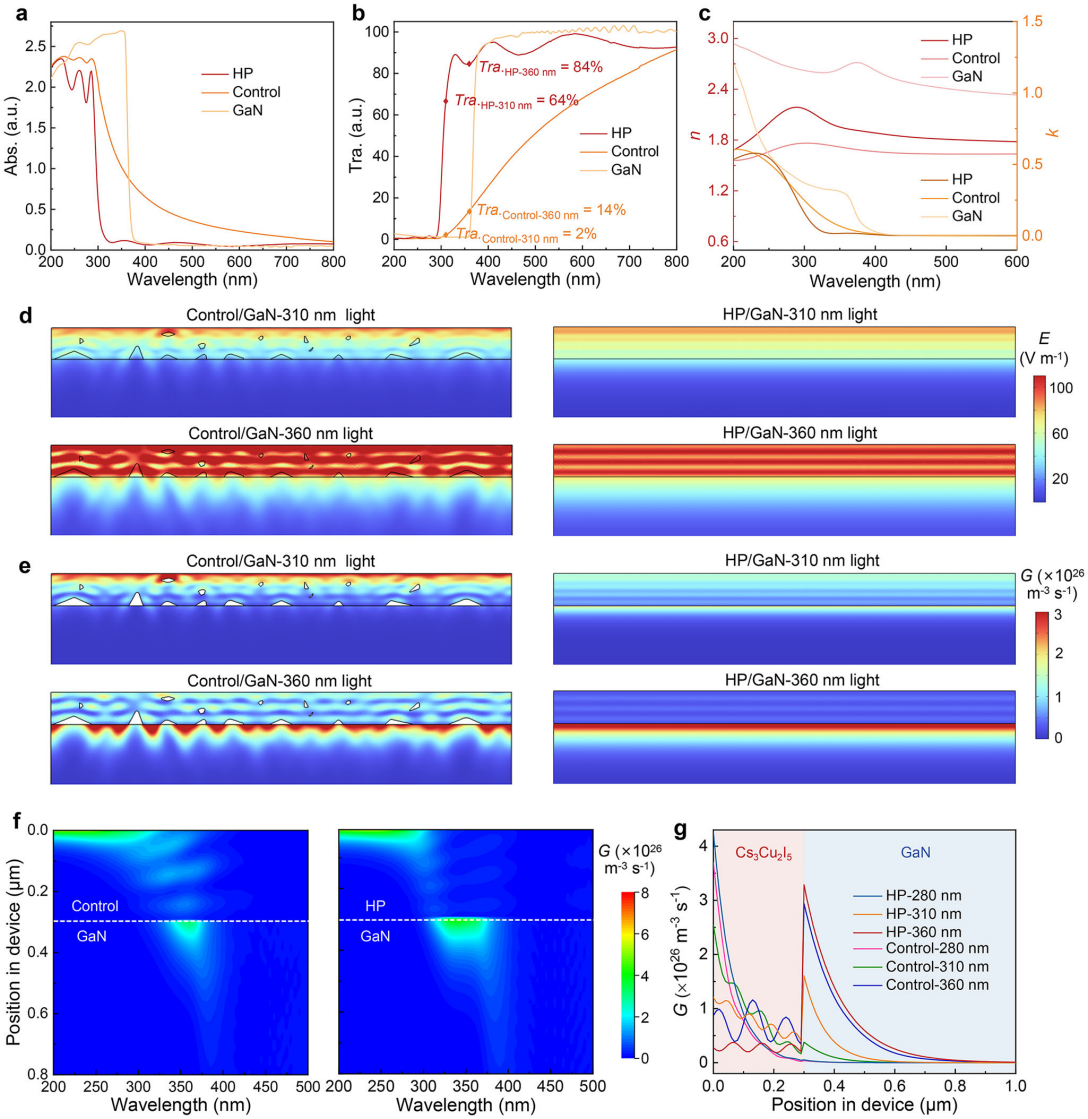
a) Absorption spectra, b) transmission spectra, and c) refractive index *n* and extinction coefficient *k* of the control films, HP films, and GaN. d) Calculated optical field distributions and e) photogenerated carrier distributions of the control device (left) and HP devices (right) at 310 and 360 nm incident light (the source injected a plane wave in the top direction). The thickness of the upper Cs_3_Cu_2_I_5_ layer is 300 nm. f) Simulated photogenerated carrier distributions for the control device (left) and HP device (right) at incident wavelengths of 200–500 nm. g) Profiles of the photogenerated carrier distributions in the control device and HP device at 280, 310, and 360 nm incident light, respectively.

To investigate the mechanisms underlying the enhanced narrowband photoresponse achieved through the HP strategy, UV−visible absorption spectra were first measured. As presented in **Figure** **3**a, the HP films show a sharper band edge cutoff compared to the control films. From the transmission spectra in Figure 3b, one can see that the transmission of light in 200−280 nm range is nearly zero for both samples, suggesting that light in this range is completely absorbed by the Cs_3_Cu_2_I_5_ films. Notably, the transmission of HP films is higher than that of the control films in the 280−375 nm range. Specifically, the transmission of HP films is 32‐fold at 310 nm and 6‐fold at 360 nm higher than the control films, suggesting that more light can penetrate the Cs_3_Cu_2_I_5_ to reach the GaN layer. The oscillatory behavior observed in the transmission and absorption spectra originates from the interference effects. When light is reflected and transmitted at the interfaces between materials with different refractive indices, the phase superposition of reflected/transmitted beams occurs. This results in constructive or destructive interference, which is typically manifested as periodic oscillatory peaks and valleys in the transmittance spectra and absorption spectra.^[^ ^63^, ^64^ ^]^

To explore the propagation characteristics of incident light, we simulated the optical field distributions for several representative incident wavelengths in both devices using the finite element analysis (FEA) method.^[^ ^65^, ^66^ ^]^ The optical constants (refractive index *n* and extinction coefficient *k*) used in the simulations are shown in Figure 3c, where the optical constants of GaN were obtained from the reported literature.^[^ ^67^ ^]^ The differences in the complex refractive index dispersion (*ñ*(*λ*) = *n*(*λ*) + i*k*(*λ*)) primarily stem from the improvements in the microstructure and the disorder of electronic states of the HP films. Hot‐pressing treatment significantly eliminates pores within the films. According to the Maxwell–Garnett effective medium theory,^[^ ^68^, ^69^ ^]^ the reduced pores in HP films lead to a notable increase in the real part of the refractive index *n*(*λ*) across the entire spectral range. Furthermore, the HP process reduces grain boundaries and defect states, effectively diminishing electronic disorder. This results in a sharper absorption edge and modifies the shape of the extinction coefficient *k*(*λ*) near the absorption edge, as supported by the findings of Urbach.^[^ ^70^ ^]^ The differences in the complex refractive index dispersion *ñ*(*λ*) influence both the light absorption properties of Cs_3_Cu_2_I_5_ films and the optical field distribution within the device. As illustrated in Figure S10 (Supporting Information), the strong absorption of UVC light (280 nm) by both the control and HP films limits the penetration depth to only 150 nm, preventing it from reaching the vicinity of the Cs_3_Cu_2_I_5_/GaN interface. As illustrated in Figure 3d, for UVB (310 nm) and UVA (360 nm) incident light, the presence of numerous pores and grain boundaries in the control films induces significant light scattering, whereas the HP films effectively mitigate this effect, resulting in a higher optical field intensity near the heterointerface compared to the control films. For visible incident light (400 nm), both Cs_3_Cu_2_I_5_ and GaN exhibit high transmission (> 90%), allowing the optical field to distribute throughout the heterojunction, despite the scattering effects present in the control films (Figure S10, Supporting Information).

To further investigate the mechanisms by which the HP strategy enhances the photoresponse of narrowband photodetectors, we simulated the photogenerated carrier distributions in the control and HP devices. As shown in Figure S11 (Supporting Information), for UVC (280 nm) incident light, both the control and HP films generate photogenerated carriers only near the surface. Although the HP annealing process reduces the defect states density in the HP films and thereby mitigates surface defect state recombination, the strong absorption for UVC light by HP films results in its absorption primarily within the top surface. Due to the limited carrier diffusion length, most photogenerated carriers fail to reach the Cs_3_Cu_2_I_5_/GaN interface to generate photocurrent.^[^ ^11^, ^21^ ^]^ As shown in Figure 3e, for UVB (310 nm) and UVA (360 nm) incident light, the HP device has more photogenerated carriers near the heterointerface, which can be attributed to the fact that the HP films effectively suppress light losses caused by scattering from the pores and grain boundaries compared to the control films. Additionally, the HP films with reduced grain boundaries could facilitate the carrier recombination, increasing the number of effective carriers reaching the electrodes, and thereby enhancing the photocurrent. For visible incident light (400 nm), the photon energy is lower than the bandgap energy of both Cs_3_Cu_2_I_5_ and GaN, which cannot contribute to the photocurrent (Figure S11, Supporting Information).

Then, we summarized the optical field and photogenerated carrier distributions within the control and HP devices under incident light wavelengths of 200–500 nm. As shown in Figure 3f,g, and Figure S12 (Supporting Information), for incident light in the 200–280 nm, the strong absorption of Cs_3_Cu_2_I_5_ limits the light penetration depth to only 50 nm for both the control films and HP films. Consequently, carriers are generated primarily near the surface of Cs_3_Cu_2_I_5_. For incident light in the 280–375 nm, reduced optical losses in the HP films enable light to penetrate the Cs_3_Cu_2_I_5_ films more effectively, generating most photogenerated carriers near the heterointerface, which favors a higher photoresponse. Notably, incident light with a wavelength exceeding 375 nm has photon energy below the bandgap energy of both materials, and therefore cannot generate photocurrent.

**Figure 4:**
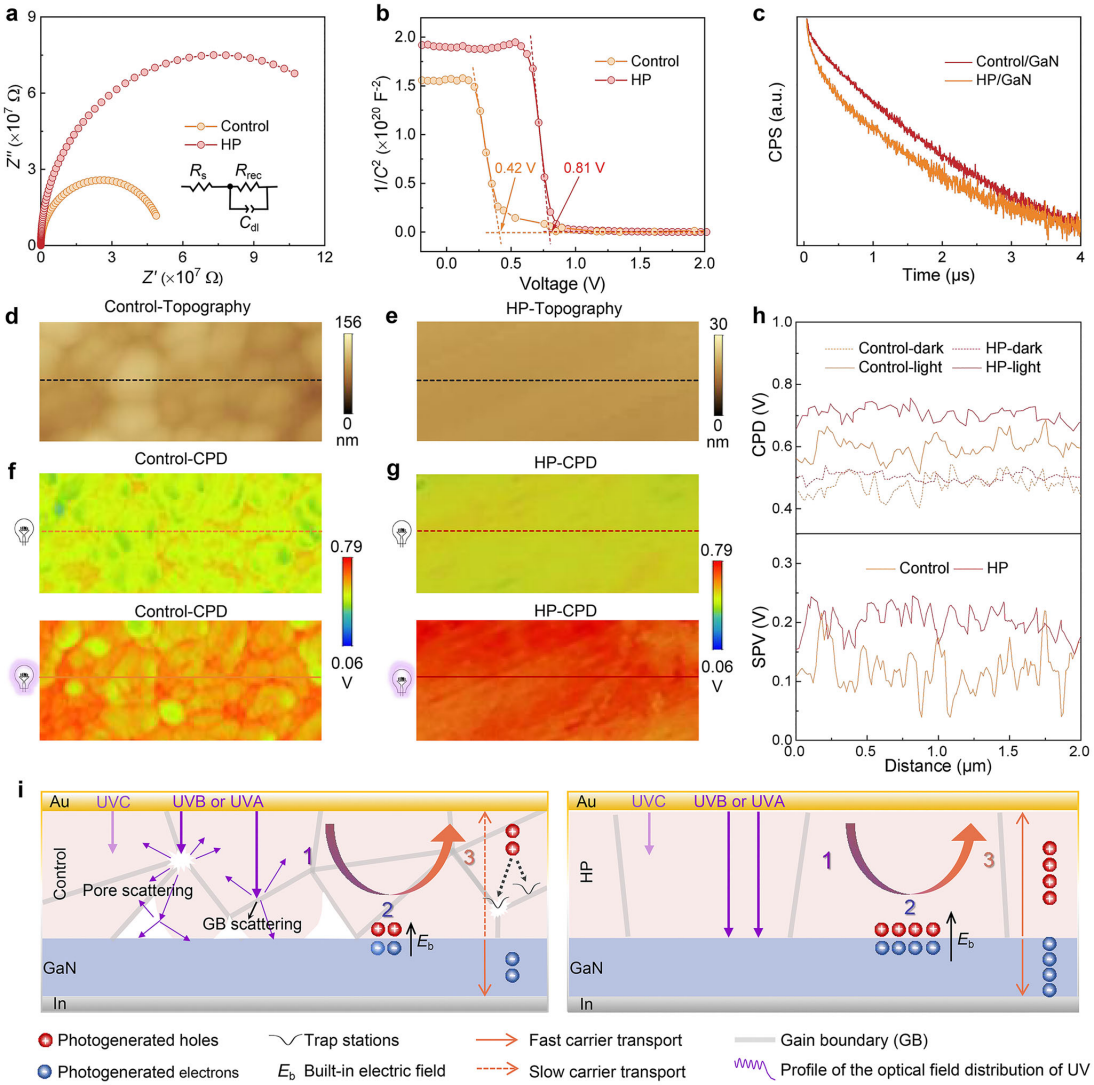
a) EIS and b) Mott–Schottky curves of the control device and HP device measured in the dark. c) Time‐resolved PL decay curves of control films/GaN and HP films/GaN heterojunctions. Topography of d) the control films and e) HP films grown on GaN. CPD of f) the control films and g) HP films grown on GaN in dark and light conditions. h) CPD profiles corresponding to the lines shown in Figure 4f,g, and surface photovoltage curves. i) The proposed mechanisms of HP‐modified photoresponse.

The interfacial properties between Cs_3_Cu_2_I_5_ and GaN were investigated by conducting the electrochemical impedance spectroscopy (EIS) and Mott–Schottky curves. **Figure** **4**a shows the Nyquist plots of the control device and HP device. The detailed fitting parameters are summarized in Table S4 (Supporting Information). The HP device demonstrates improved charge transport and subdued carrier recombination, as evidenced by smaller charge transport resistance (*R*_s_) and bigger charge recombination resistance (*R*_rec_).^[^ ^71^, ^72^ ^]^ As presented in Mott–Schottky curves shown in Figure 4b, the HP devices exhibit a higher built‐in electric field (≈0.81 V) compared with the control device (≈0.42 V), which favors the separation and transport of photogenerated carriers.^[^ ^73^ ^]^ Figure 4c presents the time‐resolved PL decay curves of control films/GaN and HP film/GaN heterojunctions. One can observe that the HP films/GaN heterojunction has a shorter *τ*_ave._ (Table S5, Supporting Information), which implies that there is a more effective and faster electron transport from Cs_3_Cu_2_I_5_ to GaN in the HP films/GaN heterojunction.

In addition, to assess the photogenerated carriers accumulation process, we measured the CPD of the control films (Figure 4d) and HP films (Figure 4e) prepared on GaN under dark and light (360 nm) conditions using KPFM. As displayed in Figure 4f,g, under dark conditions, both films show similar potentials, indicating that the HP process does not alter the work function of the Cs_3_Cu_2_I_5_ material. Upon illumination, the built‐in electric field facilitates the transport of light‐induced holes from GaN to Cs_3_Cu_2_I_5_, resulting in the accumulation of positive charges within the Cs_3_Cu_2_I_5_ layer. As shown in the CPD profiles and surface photovoltage (SPV = CPD_light_ − CPD_dark_) in Figure 4h, the HP films exhibit a higher SPV compared to the control films, demonstrating a greater accumulation of positive charges, which indicates that the photogenerated carriers are separated and transported more efficiently in the HP films/GaN heterojunction.

From the above results, we propose the relevant mechanisms to explain the HP‐enhanced narrowband photoresponse. As illustrated in Figure 4i, the generation of photocurrent involves three primary stages: 1) photons product excitons (photogenerated electron‐hole pairs) and excitons reach the depletion region; 2) the excitons are separated into free electrons and holes by the built‐in electric field; 3) the free electrons and holes are collected in opposite directions under the built‐in electric field. To achieve a narrowband photoresponse only to UVB and UVA light, the CCN effect needs to be utilized to prevent photons within the UVC range from generating photocurrent in Cs_3_Cu_2_I_5_/GaN heterojunction devices. While achieving this purpose relies on the surface defect states, and excessive defect states will degrade the device performance. In the first stage, the HP process effectively reduces the defect state density to enhance device performance and simultaneously guarantees that UVC photons cannot generate photocurrent to maintain the narrowband detection capability. Furthermore, the suppressed photon scattering within the 280–375 nm range in HP films enhances photon penetration to the heterointerface, increasing photogenerated carriers. In the second stage, the HP films establish a stronger built‐in electric field, promoting more efficient separation and transport of photogenerated carriers within the 280–375 nm range. In the third stage, improved vertical charge transport and reduced defect density in the HP films facilitate more efficient transmission and collection of photogenerated holes to the electrode. Therefore, the HP process plays a synergistic role throughout these stages in achieving faster and better responsivity while maintaining the narrowband (280–375 nm) detection capability.

**Figure 5:**
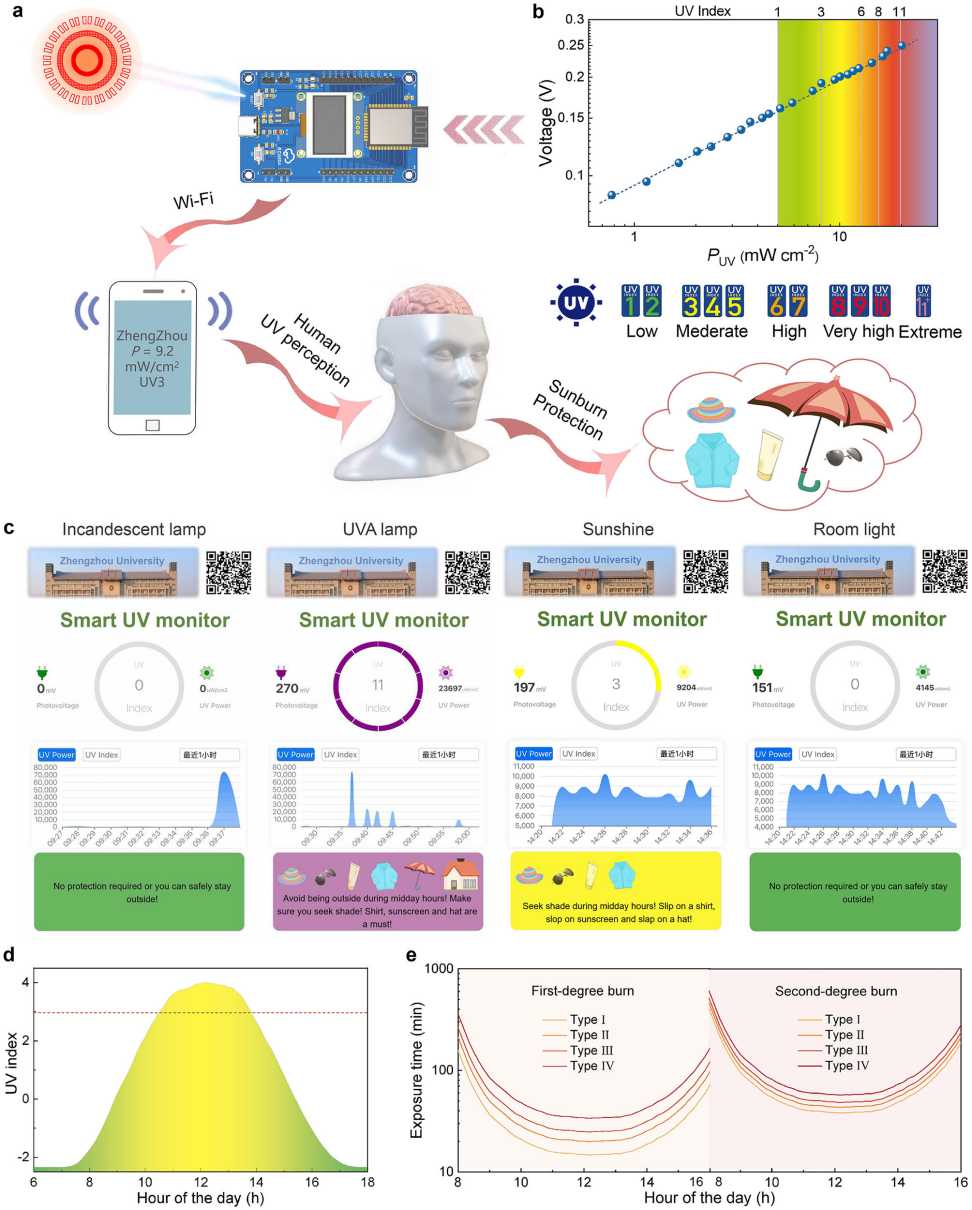
a) The working schematic of the real‐time UV radiation monitor. b) Relationship between photovoltage and UV light power, as well as the UV index. c) Screenshots of the developed APP of the real‐time UV radiation monitor under varying light sources. d) UV index trends on January 6, 2025 (sunny day). e) Exposure time under sunlight that can generate the first‐ and second‐degree sunburn depending on the skin types of humans (type I–IV).

Given that UV intensity fluctuates over time and across different locations, a real‐time UV radiation monitor that can accurately and timely provide UV information to prevent UV radiation damage becomes crucial.^[^ ^74^ ^]^ In this context, the HP device holds immense potential in real‐time UV radiation monitor applications. The HP device exhibits a remarkable selective response to the light within the UVA‐UVB range (280–375 nm) without relying on the light filters, which not only enables the device to be portable and cost‐effective but also endows it with strong resistance to interference from visible light and UVC light. In addition, the outstanding sensitivity of the HP device renders it to be readily integrated as a UV sensor module with a commercial data collector, as depicted in **Figure** **5**a. Subsequently, the real‐time data is transmitted to a self‐developed smartphone application (APP) via Wi‐Fi. Eventually, the smartphone application displays information regarding external UV radiation. Therefore, UV light, which is imperceptible to the human eye, is transformed into digital information that can be recognized by the human eye. Simultaneously, it provides corresponding protection recommendations in real‐time to safeguard humans from the risks associated with excessive UV exposure. Overall, the prepared real‐time UV radiation monitoring system comprises three main parts: an HP device serving as the UV sensor, a circuit board acting as the data processing module, and a smartphone APP functioning as the display module.

We tested the UV light power (*P*_UV_) of solar radiation using a commercial UV light power meter and obtained the photovoltage under different *P*_UV_. As shown in Figure 5b, the photovoltage exhibits a strong linear response to *P*_UV_, enabling the prepared UV radiation monitor to accurately obtain the corresponding UV light power (*P*_UV_’, mW cm^−2^) based on the measured photovoltage. In addition, UVI is ascertained by the formula of *P*_UV_’ = 1.51 + UVI × 3.55.^[^ ^75^ ^]^ To evaluate the working aptitude of the prepared UV radiation monitor, UV radiation information was measured under various light sources. As shown in Figure 5c and Video S1 (Supporting Information), under illumination from either an incandescent lamp or a UVC (265 nm) lamp, both the UV index and UV light power are 0. This result confirms that the device exhibits excellent anti‐interference performance, enabling accurate UVA and UVB light detection while remaining unaffected by visible light and solar blind light. Under the UVA (365 nm) lamp, the UV index is measured at 11, reflecting the high UV radiation intensity emitted by the UVA lamp. Under sunlight on March 12, 2025 (cloudy day), the UV index is recorded at 3. When the UV radiation monitor is moved indoors, the UV index drops to 0 due to the diffuse reflection of sunlight from the windows, and the UV light power decreases from 9.20 to 4.14 mW cm^−2^. In addition, as presented in Video S2 (Supporting Information), when the UV radiation monitor is moved from the direct sunlight to the shade of the tree on March 26, 2025 (sunny day), the shading effect of the leaves causes the UV index to drop sharply from 3 to 0, while the UV light power decreases from 8.66 to 1.78 mW cm^−2^. These results demonstrate that the developed UV radiation monitor possesses the capability to real‐time monitor both the UV light power and UV index.

Moreover, we tested and recorded the trends of UV indexes on January 6, 2025 (sunny day). As presented in Figure 5d, the developed UV radiation monitor is capable of monitoring the UV index continuously throughout the day, with data recorded at 2‐min intervals. The UV index exceeds 3 during the time from 10:53 am to 1:08 pm. As we all know, prolonged exposure to UV radiation will lead to skin sunburn. The sunburn degree depends on the UV radiation dose (*D*), which can be calculated by the formula of *D* = *P*_UV_’ × *t*_e_, where *t*_e_ is exposure time.^[^ ^75^ ^]^ As presented in Figure 5e, by using the beginning dose of first‐degree burns and second‐degree burns for skin type I–IV,^[^ ^76^, ^77^ ^]^ we calculated the exposure time for people with skin type I–IV on January 6, 2025, at Henan (34.74° N, 113.64° E) in China. The calculated exposure time allows individuals to understand the thresholds at which first‐degree and second‐degree burns could occur, helping to better assess the potential risks of sun exposure for different skin types on that specific day.

## 3 Conclusion

In summary, we have proposed an HP annealing strategy to prepare high‐quality Cs_3_Cu_2_I_5_ films with few grain boundaries, high crystallinity, and vertically aligned grains. Compared to the control films, the HP films possess reduced defect density, achieving an optimal balance between the narrowband detection capability and overall device performance. Benefiting from the enhanced transmissivity in the 280–375 nm, higher built‐in electric field, and superior vertical transport of the HP films, the integration of HP films with GaN enables a faster and more efficient narrowband (280–375 nm) photoresponse. Finally, the photodetectors culminate in the successful implementation of real‐time UV radiation monitors to help prevent diseases caused by excessive UV radiation. It is believed that this work provides new insights into the manufacture of high‐performance self‐powered narrowband UV photodetectors, making practical applications of them in real‐time UV radiation monitors a real possibility.

## 4 Experimental Section

### Materials

Cesium iodide (CsI, 99.99%) and copper iodide (CuI, 99.99%) were purchased from Xi´an Polymer Light Technology Corp. N,N‐dimethylformamide (DMF, 99%), Dimethyl Sulfoxide (DMSO, 99%), and toluene (Sigma–Aldrich, 99.7%) were purchased from Sigma–Aldrich. All chemicals and solvents were used as received without further purification.

### Preparation of the Cs_3_Cu_2_I_5_ Films

First, CsI and CuI in a 3:2 molar ratio were dissolved in the mixed solution of DMF and DMSO with the volume ratio 1:1, stirred magnetically at 25 °C for 12 h, and filtered through a 0.22 µm polyvinyl fluoride filter to obtain 0.5 mol L^−1^ solution. Then, the precursor solution was spin‐coated onto cleaned substrates at 500 rpm for 5 s, and then at 3000 rpm for 50 s. During the last 26 s, toluene was added to accelerate crystallization. For preparing the control films, the samples were annealed at 150 °C for 1 h. For preparing the HP films, the samples were placed in a HP machine at 150 °C, covered with a silicon wafer, and imposed 180 MPa press to carry out HP annealing for 1 h. The Cs_3_Cu_2_I_5_ films were fabricated in an argon‐filled glovebox (with both H_2_O and O_2_ levels maintained below 0.1 ppm).

### Device Fabrication

Au electrodes (50 nm) and In electrodes (100 nm) were deposited by magnetron sputtering on the Cs_3_Cu_2_I_5_ films and GaN, respectively. The active area of the device was 0.79 mm^2^.

### Materials and Device Characterizations

The morphology was examined by field‐emission SEM (Jeol‐7500F, 15 keV). The pressure was applied by a HP machine (MITR, PCH‐600C). The crystallinity characterization was performed by XRD (Panalytical, X'Pert Pro). The contact potential difference was performed by frequency‐modulated high‐resolution AFM (Shimadzu, SPM‐9700). The absorption spectrum was tested by an UV–visible spectrophotometer (Shimadzu, UV‐3600). The optical constants were tested by a spectroscopic ellipsometer (Eoptics, SE‐VE). The PL spectra were performed by spectrofluorometer (Horiba, Fluorolog‐3), and the PL lifetimes were tested by pulsed NanoLED (Horiba, 280 nm). UV light power was measured by a commercial UV light power meter (Lianhuicheng Technology Co., Ltd., LH‐125). The photoelectric tests of the photodetector were executed via a testing system including 360 nm light sources (Changchun New Industries, UV‐F‐360), a grating spectrograph (Shanghai Precision Instruments Co., Ltd., Omni‐λ300), a digital source meter (Keithley, 2636B), and an optical chopper (SRS, SR540) in air.

### Surface Energy Calculations

All the calculations were carried out using Materials Studio. A slab model was established based on the crystal structure of Cs_3_Cu_2_I_5_. Geometry and energy optimization were carried out using the PBE functional within the GGA to handle the exchange‐correlation energy. The convergence criteria for energy and force were set to 10^−4^ eV and 0.005 eV Å^−1^, respectively, with a *k*‐point grid of 1×1×2. To avoid interactions between adjacent molecular layers, a vacuum layer of 15 Å was applied. The surface energy can be calculated using the following equation of *E*_surface_ = (*E*_slab_ − *nE*_bulk_)/*A*, where *E*_slab_ is the total energy of the surface slab model, *n* is the number of formula units of the bulk phase contained in the slab model, *E*_bulk_ is the energy of a unit in the bulk phase, and *A* represents the surface area of the surface slab, respectively.

### Optical Field and Photogenerated Carrier Distribution Simulation

Optical field and photogenerated carrier distribution simulations were performed using the COMSOL Multiphysics simulation tool (Wave Optics Module) by solving Maxwell's equations in the frequency domain.^[^ ^65^, ^66^ ^]^ During the simulation, a 2D geometric model of the devices were constructed. The modeling of the control films and the HP films was derived from morphological characterizations obtained from cross‐sectional and top‐view SEM images shown in Figure 1b−e. GaN was modeled as a dense and flat single‐crystalline layer. As illustrated in Figure S13 (Supporting Information), the physical domains from top to bottom consist of air, Au, Cs_3_Cu_2_I_5_, GaN, and Al_2_O_3_. The optical constants (*n* and *k*) of the Cs_3_Cu_2_I_5_ films were obtained using a spectroscopic ellipsometer (Eoptics, SE‐VE). The optical constants for air, Au, and Al_2_O_3_ were obtained from the material library of the simulation software. Additionally, Perfectly Matched Layers (PMLs) were applied at the top and bottom boundaries to simulate an infinitely thick Al_2_O_3_ substrate and air. The lateral boundaries of all domains were assigned Floquet periodic boundary conditions to approximate a laterally infinite dimension, where the Floquet periodic *k* vector was derived from the periodic ports. Two periodic ports were set at the upper interface of the air domain and the upper interface of the bottom PML domain. The wave excitation of the port at the top air boundary was enabled, while that at the top interface of the bottom PML was disabled. The incident light power density was set to 10 mW cm^−2^. A parameterized wavelength sweep was performed from 200 to 500 nm to simulate vertical illumination from the air side onto the devices. The distribution of photogenerated carrier rate *G* can be calculated by using the following equation of $G=real|\nabla \cdot P_{\mathrm{op}}|\times \frac{\eta }{h\lambda }$, where *η* is the quantum yield (the number of electron‐hole pairs generated per incident photon) assumed to be equal to the unity, *P*_op_ is a time‐varying power flow under the light field, and *λ* is the wavelength of the incident light, respectively.

## Conflict of Interest

The authors declare no conflict of interest.

## Supporting information



Supporting Information

Supplemental Video 1

Supplemental Video 2

## Data Availability

The data that support the findings of this study are available from the corresponding author upon reasonable request.

## References

[1] Radiation: Ultraviolet (UV) Radiation, World Health Organization , https://www.who.int/news‐room/questions‐and‐answers/item/radiation‐ultraviolet‐(uv) (accessed: December 2024).

[2] Ultraviolet Radiation, World Health Organization , https://www.who.int/news‐room/fact‐sheets/detail/ultraviolet‐radiation (accessed: December 2024.

[3] M. Saraiya , K. Glanz , P. A. Briss , P. Nichols , C. White , D. Das , S. J. Smith , B. Tannor , A. B. Hutchinson , K. M. Wilson , N. Gandhi , N. C. Lee , B. Rimer , R. C. Coates , J. F. Kerner , R. A. Hiatt , P. Buffler , P. Rochester , Am. J. Prev. Med. 2024, 27, 422. 10.1016/j.amepre.2004.08.009 15556744

[4] X. Huang , A. N. Chalmers , Ann. Biomed. Eng. 2021, 49, 964. 33432511 10.1007/s10439-020-02710-x

[5] M. Gröbner , J. Gröbner , G. Hülsen , Photochem. Photobiol. Sci. 2015, 14, 352. 25410623 10.1039/c4pp00324a

[6] A. R. Webb , L. Kline , M. F. Holick , J. Clin. Endocrinol. Metab. 1988, 67, 373. 2839537 10.1210/jcem-67-2-373

[7] Z. Wang , J. Xiong , J. Zhou , Z. Han , Water Environ. Res. 2025, 97, 70049. 10.1002/wer.70049 40088081

[8] X. Tang , G. F. Wu , K. W. C. Lai , J. Mater. Chem. C 2017, 5, 362.

[9] Y. Wu , X. J. Sun , Y. P. Jia , D. B. Li , Chin. Phys. B 2018, 27, 126101.

[10] Y. Xu , R. Li , S. Bai , Y. Li , Z. Jia , Y. Yang , E. Cui , F. Yao , D. Wang , C. Lei , Q. Lin , Adv. Funct. Mater. 2022, 33, 2212523.

[11] Q. Lin , A. Armin , P. L. Burn , P. Meredith , Nat. Photonics 2015, 175, 687.

[12] A. Armin , R. D. Jansen‐van Vuuren , N. Kopidakis , P. L. Burn , P. Meredith , Nat. Commun. 2015, 6, 6343. 25721323 10.1038/ncomms7343

[13] Y. Han , J. Fang , H. Zhang , Y. Sun , Y. Yuan , X. Chen , M. Jia , X. Li , H. Gao , Z. Shi , Energy Mater. Adv. 2024, 5, 0116.

[14] Y. Fang , Q. Dong , Y. Shao , Y. Yuan , J. Huang , Nat. Photonics 2015, 9, 679.

[15] M. I. Saidaminov , M. A. Haque , M. Savoie , A. L. Abdelhady , N. Cho , I. Dursun , U. Buttner , E. Alarousu , T. Wu , O. M. Bakr , Adv. Mater. 2016, 28, 8144. 27390113 10.1002/adma.201601235

[16] J. Wang , S. Xiao , W. Qian , K. Zhang , J. Yu , X. Xu , G. Wang , S. Zheng , S. Yang , Adv. Mater. 2021, 33, 2005557. 10.1002/adma.202005557 33300215

[17] J. Ma , M. Zhang , H. Jiang , X. Chen , W. Di , X. Li , Y. Zhang , C. Shan , Z. Shi , Nano Today 2023, 52, 101970.

[18] J. Ma , X. Xia , S. Yan , Y. Li , W. Liang , J. Yan , X. Chen , D. Wu , X. Li , Z. Shi , ACS Appl. Mater. Interfaces 2021, 13, 15409. 33779137 10.1021/acsami.1c00387

[19] Z. X. Zhang , C. Li , Y. Lu , X. W. Tong , F. X. Liang , X. Y. Zhao , D. Wu , C. Xie , L. B. Luo , J. Phys. Chem. Lett. 2019, 10, 5343. 31452370 10.1021/acs.jpclett.9b02390

[20] F. Zeng , Y. Guo , W. Hu , Y. Tan , X. Zhang , J. Feng , X. Tang , ACS Appl. Mater. Interfaces 2020, 12, 23094. 32336082 10.1021/acsami.0c03106

[21] Y. Li , Z. Shi , W. Liang , L. Wang , S. Li , F. Zhang , Z. Ma , Y. Wang , Y. Tian , D. Wu , X. Li , Y. Zhang , C. Shan , X. Fang , Mater. Horiz. 2020, 7, 530.

[22] X. Zhou , Z. Lu , L. Zhang , Q. Ke , Nano Energy 2023, 117, 108908.

[23] R. Heiderhoff , T. Haeger , N. PourdavoudP , T. Hu , M. Al‐Khafaji , A. Mayer , Y. W. Chen , H. C. Scheer , T. Riedl , J. Phys. Chem. C 2017, 121, 28306.

[24] X. Fu , N. Dong , G. Lian , S. Lv , T. Zhao , Q. Wang , D. Cui , C. P. Wong , Nano Lett. 2018, 18, 1213. 29389136 10.1021/acs.nanolett.7b04809

[25] H. Jiang , H. Ji , Z. Ma , D. Yang , J. Ma , M. Zhang , X. Li , M. Wang , Y. Li , X. Chen , D. Wu , X. Li , C. Shan , Z. Shi , Nat. Commun. 2019, 10, 1989.31040278

[26] N. Pourdavoud , T. Haeger , A. Mayer , P. J. Cegielski , A. L. Giesecke , R. Heiderhoff , S. Olthof , S. Zaefferer , I. Shutsko , A. Henkel , D. Becker‐Koch , M. Stein , M. Cehovski , O. Charfi , H. H. Johannes , D. Rogalla , M. C. Lemme , M. Koch , Y. Vaynzof , K. Meerholz , W. Kowalsky , H. C. Scheer , P. Gorrn , T. Riedl , Adv. Mater. 2019, 31, 1903717. 10.1002/adma.201903717 31402527

[27] R. Yang , D. Yang , M. Wang , F. Zhang , X. Ji , M. Zhang , M. Jia , X. Chen , D. Wu , X. Li , Y. Zhang , Z. Shi , C. Shan , ACS Appl. Mater. Interfaces 2018, 10, 8393.29488378

[28] L. Huang , Z. Xing , X. L. Tang , D. X. Li , X. C. Meng , X. T. Hu , T. Hu , Y. W. Chen , J. Mater. Chem. A 2021, 9, 16178.

[29] L. Huang , B. Yu , F. Zhu , W. Tian , G. Lian , T. Zhang , H. Yu , D. Cui , Q. Wang , Q. Meng , C.‐P. Wong , Nano Energy 2022, 92,106719.

[30] J. Ma , M. Zhang , C. Dun , H. Ji , Z. Ma , X. Chen , X. Li , D. Wu , Z. Shi , Adv. Funct. Mater. 2025, 35, 2414943.

[31] Y. Chen , H. Zeng , P. Ma , G. Chen , J. Jian , X. Sun , X. Li , H. Wang , W. Yin , Q. Jia , G. Zou , Angew. Chem., Int. Ed. 2020, 60, 2629. 10.1002/anie.202011853 33047467

[32] B. Y. Deng , H. F. Li , Z. H. Liao , Z. R. Zhou , F. Wang , J. Mater. Chem. C 2024, 12, 14165.

[33] R. German , Sintering: From Empirical Observations to Scientific Principles, Press of Butterworth‐Heinemann Publishing House of Electronics Industry, UK 2014.

[34] W. Li , H. Li , W. Li , B. Li , T. Lu , X. Feng , C. Guo , H. Zhang , H. Wei , B. Yang , Adv. Mater. 2022, 34, 2108020. 10.1002/adma.202108020 34865244

[35] W. Tan , Y. Xiao , C. Zhou , X. Jin , S. Zhu , M. Han , Z. Tang , Y. Zhang , Z. Su , T. Chen , Q. Chen , Q. Liang , W. Chen , Y. Jiang , Adv. Funct. Mater. 2024, 34, 2406839.

[36] M. A. Haque , A. N. Gandi , R. Mohanraman , Y. Weng , B. Davaasuren , A.‐H. Emwas , C. Combe , D. Baran , A. Rothenberger , U. Schwingenschlögl , H. N. Alshareef , S. Dong , T. Wu , Adv. Funct. Mater. 2019, 29, 1809166.

[37] L. Wang , Z. Shi , Z. Ma , D. Yang , F. Zhang , X. Ji , M. Wang , X. Chen , G. Na , S. Chen , D. Wu , Y. Zhang , X. Li , L. Zhang , C. Shan , Nano Lett. 2020, 20, 3568. 32243171 10.1021/acs.nanolett.0c00513

[38] M. Wang , X. Chen , F. Zhang , Z. Ma , X. Ji , S. Cheng , G. Pan , D. Wu , X.‐J. Li , Y. Zhang , C. Shan , Z. Shi , ACS Nano 2024, 18, 30421. 39455431 10.1021/acsnano.4c07641

[39] C. S. Jiang , M. Yang , Y. Zhou , B. To , S. U. Nanayakkara , J. M. Luther , W. Zhou , J. J. Berry , J. van de Lagemaat , N. P. Padture , K. Zhu , M. M. Al‐Jassim , Nat. Commun. 2015, 6, 8397. 26411597 10.1038/ncomms9397 PMC4598624

[40] J. S. Yun , A. Ho‐Baillie , S. Huang , S. H. Woo , Y. Heo , J. Seidel , F. Huang , Y. B. Cheng , M. A. Green , J. Phys. Chem. Lett. 2015, 6, 875. 26262666 10.1021/acs.jpclett.5b00182

[41] J. L. Garrett , E. M. Tennyson , M. Hu , J. Huang , J. N. Munday , M. S. Leite , Nano Lett. 2017, 17, 2554. 28226210 10.1021/acs.nanolett.7b00289

[42] Y. Fang , A. Armin , P. Meredith , J. Huang , Nat. Photonics 2019, 13, 1.

[43] X. Zhan , X. Zhang , Z. Liu , C. Chen , L. Kong , S. Jiang , S. Xi , G. Liao , X. Liu , ACS Appl. Mater. Interfaces 2021, 13, 45744. 34545739 10.1021/acsami.1c15013

[44] R. Zhuo , Y. Wang , D. Wu , Z. Lou , Z. Shi , T. Xu , J. Xu , Y. Tian , X. Li , J. Mater. Chem. C 2018, 6, 299.

[45] C. Wei , J. Xu , S. Shi , Y. Bu , R. Cao , J. Chen , J. Xiang , X. Zhang , L. Li , J. Colloid Interface Sci. 2020, 577, 279. 32485411 10.1016/j.jcis.2020.05.077

[46] D. Wu , M. Xu , L. Zeng , Z. Shi , Y. Tian , X. J. Li , C. X. Shan , J. Jie , ACS Nano 2022, 16, 5545. 35324154 10.1021/acsnano.1c10181

[47] Z. Wang , R. Yu , C. Pan , Z. Li , J. Yang , F. Yi , Z. L. Wang , Nat. Commun. 2015, 6, 8401. 26403916 10.1038/ncomms9401 PMC4598631

[48] L. H. Zeng , Q. M. Chen , Z. X. Zhang , D. Wu , H. Yuan , Y. Y. Li , W. Qarony , S. P. Lau , L. B. Luo , Y. H. Tsang , Adv. Sci. 2019, 6, 1901134. 10.1002/advs.201901134 PMC6774060 31592422

[49] L. Zheng , X. Deng , Y. Wang , J. Chen , X. Fang , L. Wang , X. Shi , H. Zheng , Adv. Funct. Mater. 2020, 30, 2001604.

[50] Y. Peng , J. Lu , X. Wang , W. Ma , M. Que , Q. Chen , F. Li , X. Liu , W. Gao , C. Pan , Nano Energy 2022, 94, 106945.

[51] R. Zhuo , D. Wu , Y. Wang , E. Wu , C. Jia , Z. Shi , T. Xu , Y. Tian , X. Li , J. Mater. Chem. C 2018, 6, 10982.

[52] D. Guo , Y. Su , H. Shi , P. Li , N. Zhao , J. Ye , S. Wang , A. Liu , Z. Chen , C. Li , W. Tang , ACS Nano 2018, 12, 12827. 30485072 10.1021/acsnano.8b07997

[53] X. Chen , Y. Xu , D. Zhou , S. Yang , F. F. Ren , H. Lu , K. Tang , S. Gu , R. Zhang , Y. Zheng , J. Ye , ACS Appl. Mater. Interfaces 2017, 9, 36997. 28975779 10.1021/acsami.7b09812

[54] V. Kuryatkov , A. Chandolu , B. Borisov , G. Kipshidze , K. Zhu , S. Nikishin , H. Temkin , M. Holtz , Appl. Phys. Lett. 2003, 82, 1323.

[55] T. M. H. Nguyen , S. Kim , C. W. Bark , J. Mater. Chem. A 2021, 9, 1269.

[56] W. Liang , Z. Shi , Y. Li , J. Ma , S. Yin , X. Chen , D. Wu , Y. Tian , Y. Tian , Y. Zhang , X. Li , C. Shan , ACS Appl. Mater. Interfaces 2020, 12, 37363. 32814386 10.1021/acsami.0c10323

[57] Z. Lin , T. Lin , T. Lin , X. Tang , G. Chen , J. Xiao , H. Wang , W. Wang , G. Li , Appl. Phys. Lett. 2023, 122, 131101.

[58] B. Zhao , F. Wang , H. Chen , L. Zheng , L. Su , D. Zhao , X. Fang , Adv. Funct. Mater. 2017, 27, 1700264.

[59] H. Sun , W. Tian , F. Cao , J. Xiong , L. Li , Adv. Mater. 2018, 30, 1706986. 10.1002/adma.201706986 29638010

[60] M. Wang , P. Zeng , Z. Wang , M. Liu , Adv. Sci. 2020, 7, 1903662. 10.1002/advs.201903662 PMC7284202 32537411

[61] T. Cossuet , J. Resende , L. Rapenne , O. Chaix‐Pluchery , C. Jiménez , G. Renou , A. J. Pearson , R. L. Z. Hoye , D. Blanc‐Pelissier , N. D. Nguyen , E. Appert , D. Muñoz‐Rojas , V. Consonni , J. L. Deschanvres , Adv. Funct. Mater. 2018, 28, 1803142.

[62] UV Enhanced 100% QE Photodiodes, OSI Optoelectronics, https://osioptoelectronics.com/products/photodetectors/uv‐enhanced‐100‐qe, (accessed: December 2024).

[63] L. A. Pettersson , L. Roman , O. Inganäs , J. Appl. Phys. 1999, 86, 487.

[64] J. Ma , F. Zhang , Y. Xing , H. Jiang , Y. Tian , H. Ji , X. Chen , D. Wu , L. Zeng , X. Li , C. Shan , Z. Shi , Adv. Sci. 2025, 12, 2503498. 10.1002/advs.202503498 PMC12302572 40349180

[65] J. M. Jin , J. Q. Yin , (translator), Theory and Computation of Electromagnetic Fields, Press of Publishing House of Electronics Industry, 2nd ed., China 2018.

[66] O. C. Zienkiewicz , R. L. Taylor , J. Z. Zhu , The Finite Element Method: Its Basis and Fundamentals, Press of Butterworth‐Heinemann, UK 2013.

[67] A. B. Djurišić , E. H. Li , J. Appl. Phys. 1999, 85, 2848.

[68] G. J. C. Maxwell , Phil. Trans. R. Soc. A 1904, 203, 385.

[69] Z. Yang , D. Zhu , D. Lu , M. Zhao , N. Ning , Y. Liu , Opt. Quant. Electron. 2003, 35, 1133.

[70] F. Urbach , Phys. Rev. 1953, 92, 1324.

[71] H. Liu , L. Wang , R. Li , B. Shi , P. Wang , Y. Zhao , X. Zhang , ACS Energy Lett. 2021, 6, 2907.

[72] C. Cheng , Y. Yao , L. Li , Q. Zhao , C. Zhang , X. Zhong , Q. Zhang , Y. Gao , K. Wang , Nano Lett. 2023, 23, 8850. 37748018 10.1021/acs.nanolett.3c01769

[73] Z. Ma , X. Ji , S. Lin , X. Chen , D. Wu , X. Li , Y. Zhang , C. Shan , Z. Shi , X. Fang , Adv. Mater. 2023, 35, 2300731. 10.1002/adma.202300731 36854310

[74] Global Solar UV Index: A Practical Guide, World Health Organization https://www.who.int/publications/i/item/9241590076. (accessed: May 2024)

[75] J. F. Sanchez‐Perez , D. Vicente‐Agullo , M. Barbera , E. Castro‐Rodriguez , M. Canovas , Sci. Rep. 2019, 9, 733. 30679563 10.1038/s41598-018-36850-x PMC6345802

[76] J. F. Sánchez‐Pérez , E. González‐Ferradás , F. Díaz Alonso , D. Palacios‐García , M. V. Mínguez‐Cano , J. A. Bautista‐Cotorruelo , Process Saf. Environ. Prot. 2010, 8, 109.

[77] A. M. Stoll , L. C. Greene , J. Appl. Physiol. 1952, 14, 373. 10.1152/jappl.1959.14.3.373 13654166

