# Supplementary material for: Hot‐Pressing Annealing‐Induced Light Utilization Enhancement and Crystallinity Optimization Enable High‐Performance Narrowband Ultraviolet Photodetectors for Real‐Time Ultraviolet Radiation Monitors
Source: Adv Sci (Weinh). 2025 Oct 6;12(48):e13795. doi: 10.1002/advs.202513795 (PMC12752576; doi:10.1002/advs.202513795)
Supplement: Supplementary file 1 — Supporting Information [file ADVS-12-e13795-s003.doc]

Copyright WILEY-VCH Verlag GmbH & Co. KGaA, 69469 Weinheim, Germany, 2025.

Supporting Information

**Hot-Pressing Annealing-Induced Light Utilization Enhancement and Crystallinity Optimization Enable High-Performance Narrowband Ultraviolet Photodetectors for Real-Time Ultraviolet Radiation Monitors**

*Jingli Ma*, *Junhao Zhu*, *Conghui Dun*, *Hao Wang*, *Bangbang Yang*, *Mengyao Zhang*, *Lejin Li, Huifang Ji, Yanbing Han, Ying Liu, Di Wu, Xinjian Li, Chongxin Shan, and Zhifeng Shi**

Dr. Jingli Ma, Dr. Junhao Zhu, Dr. Conghui Dun, Dr. Hao Wang, Dr. Bangbang Yang, Dr. Mengyao Zhang, Prof. Lejin Li, Prof. Huifang Ji, Prof. Yanbing Han, Prof. Ying Liu, Prof. Di Wu, Prof. Xinjian Li, Prof. Chongxin Shan, Prof. Zhifeng Shi
Key Laboratory of Materials Physics of Ministry of Education, School of Physics, Zhengzhou University, Daxue Road 75, Zhengzhou 450052, China

E-mail: shizf@zzu.edu.cn

Keywords: hot-pressing, Cs_3_Cu_2_I_5_, narrowband ultraviolet photodetector, optical field distributions, ultraviolet radiation monitor


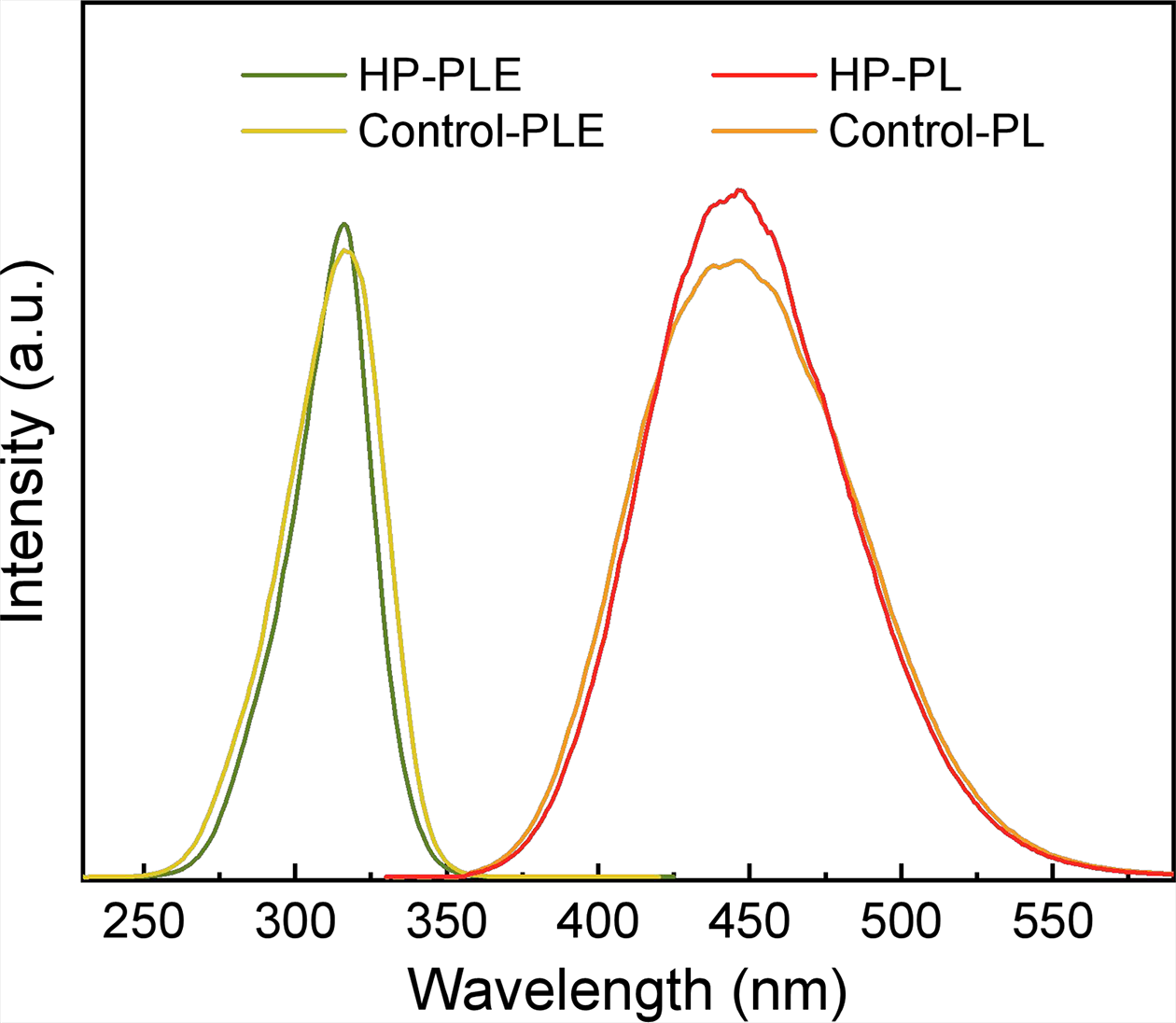


**Figure S1.** Steady-state PL and PLE spectra of the HP films and control films.


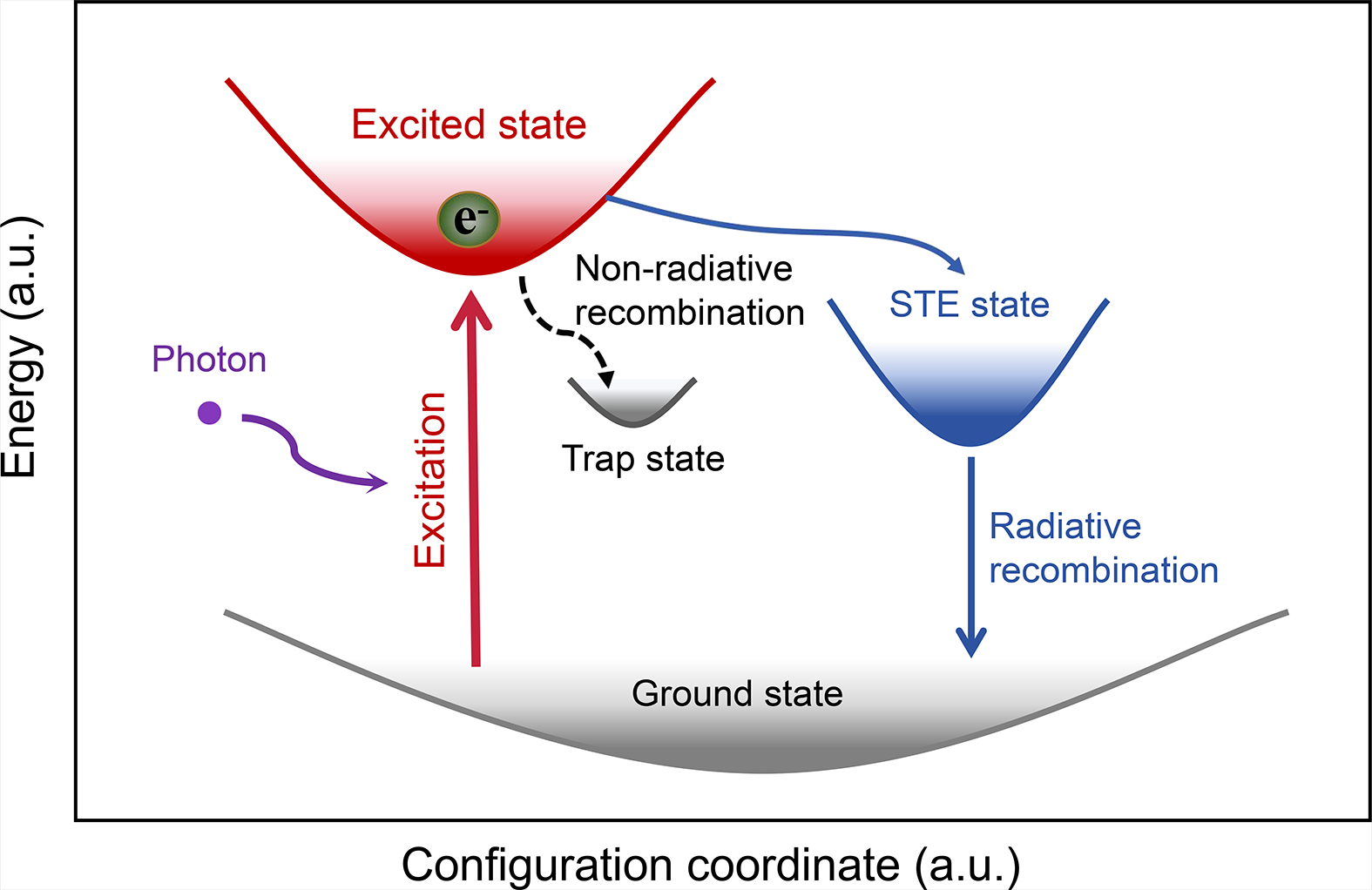


**Figure S2.** Schematic illustration of the recombination mechanism in Cs_3_Cu_2_I_5_ films.


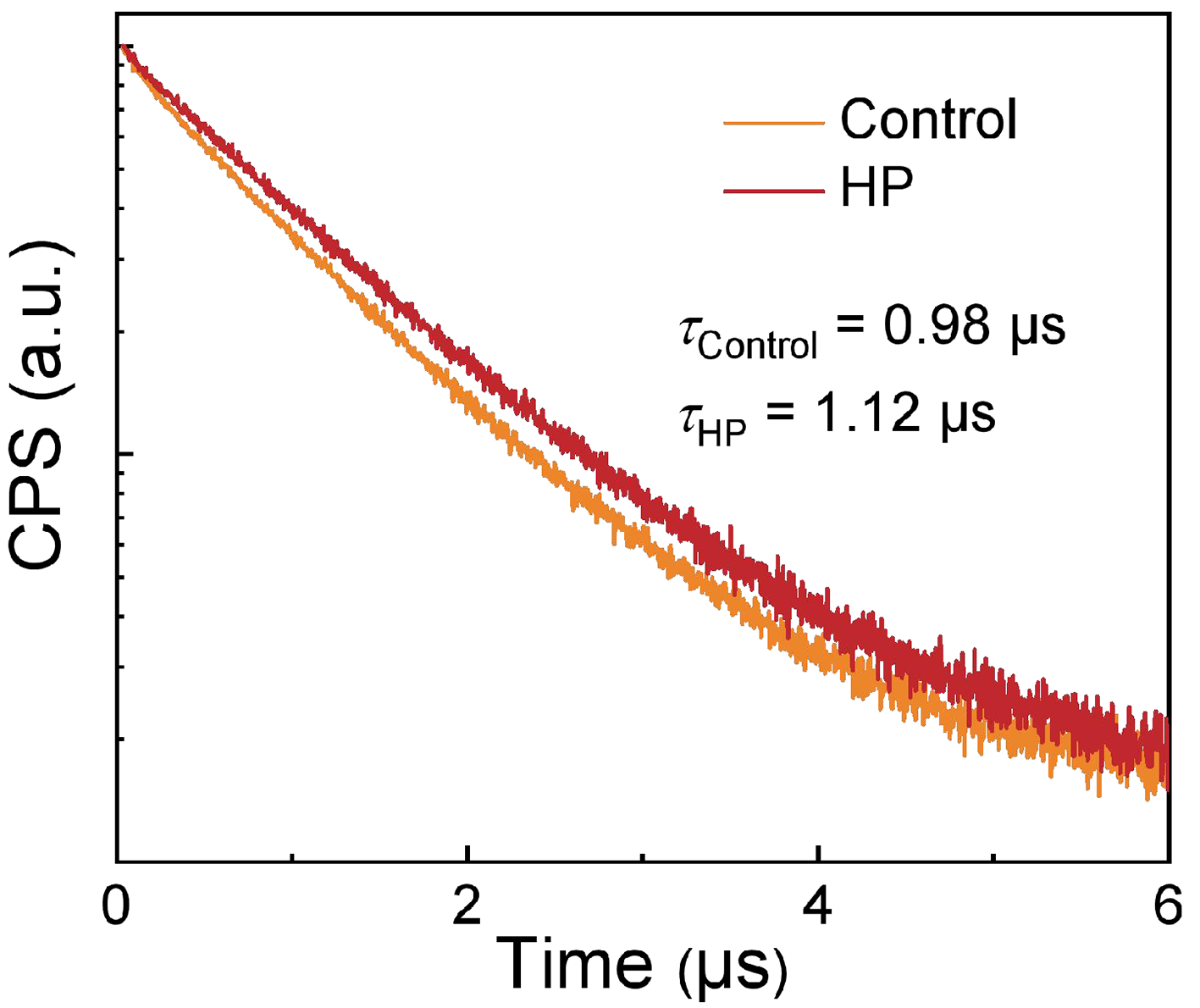


**Figure S3.** Time-resolved PL spectra of the control and HP films.


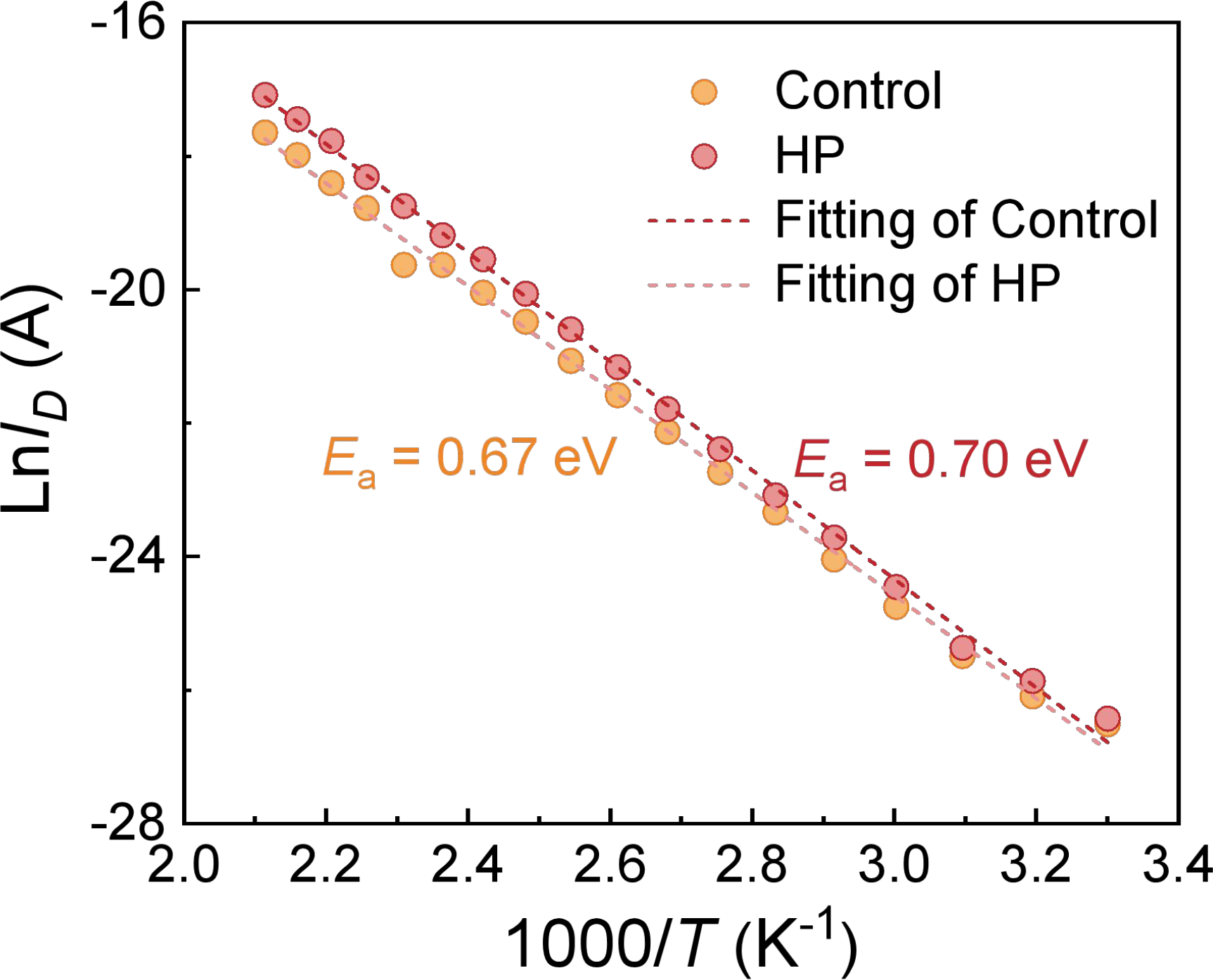


**Figure S4.** Ln*I*_D_ *versus* *T*^−1^ curves and the fitting curves using the Arrhenius equation of the control and HP films.

**
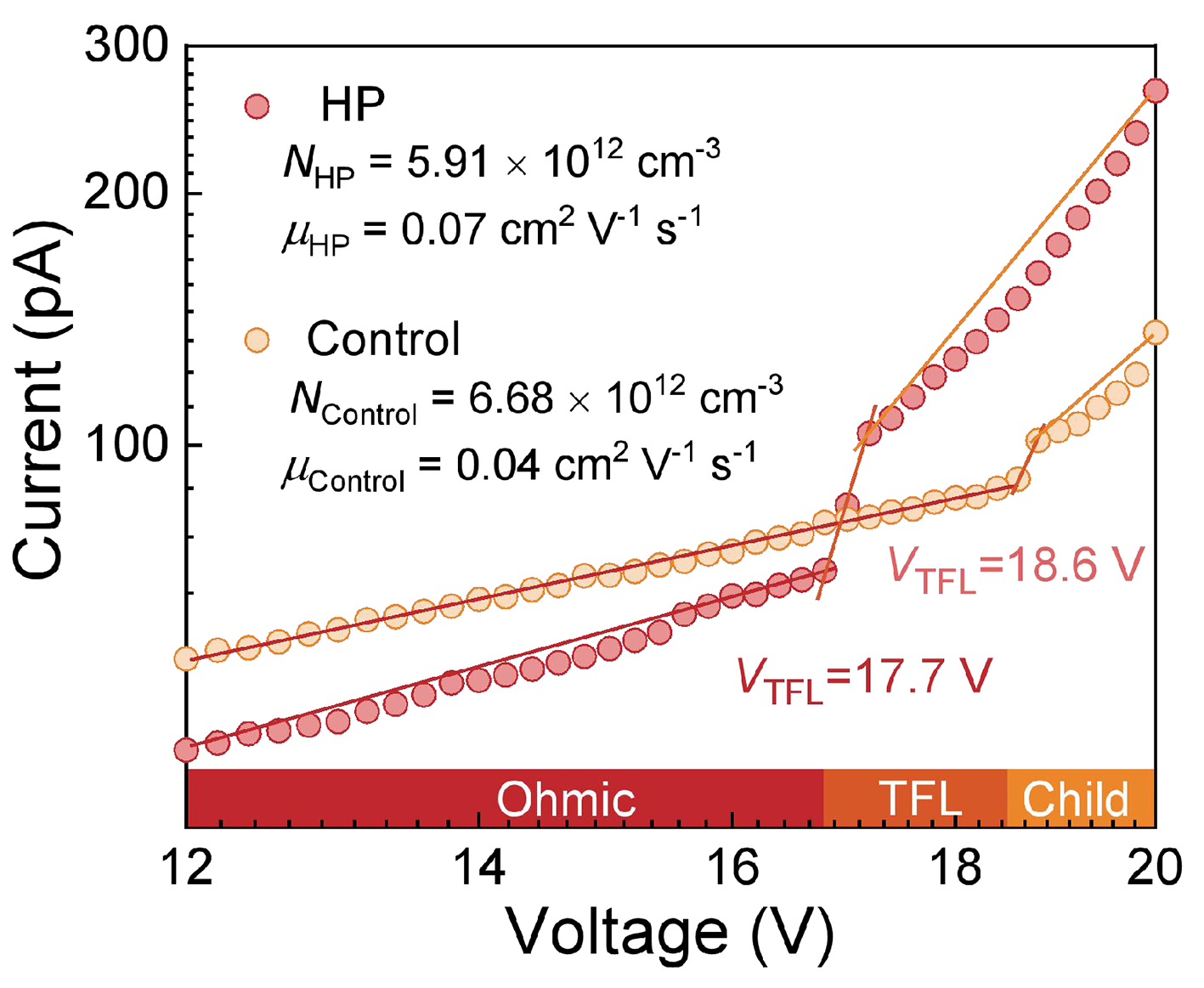
**

**Figure S5.** SCLC measurements of the control and HP films.


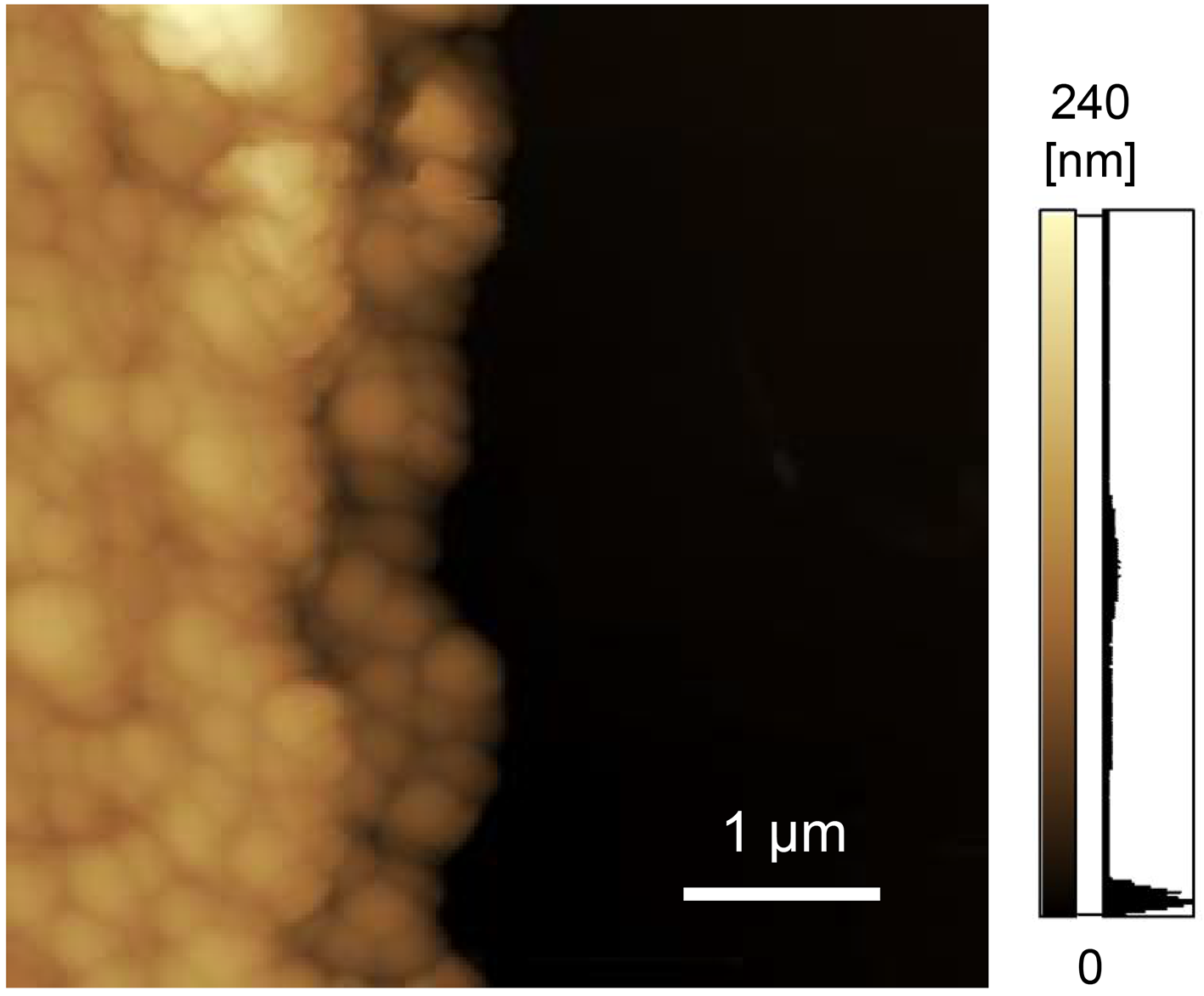


**Figure S6.** AFM image of the Cs_3_Cu_2_I_5_/GaN heterojunction.

**
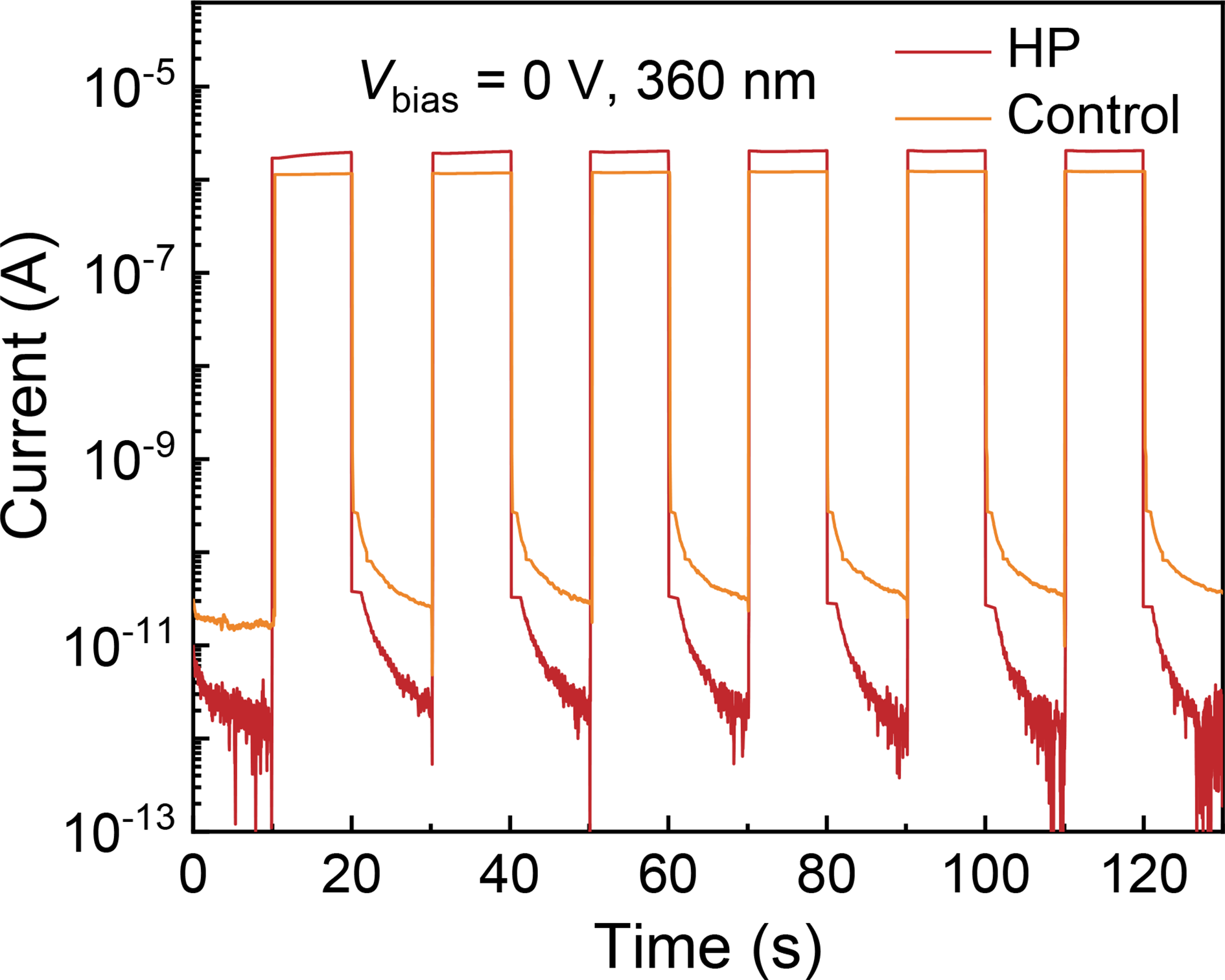
**

**Figure S7.** *I*–*t* curves of the control device and HP device under 360 nm light excitation with a light power of 12 mW cm^–2^ at zero bias.

.
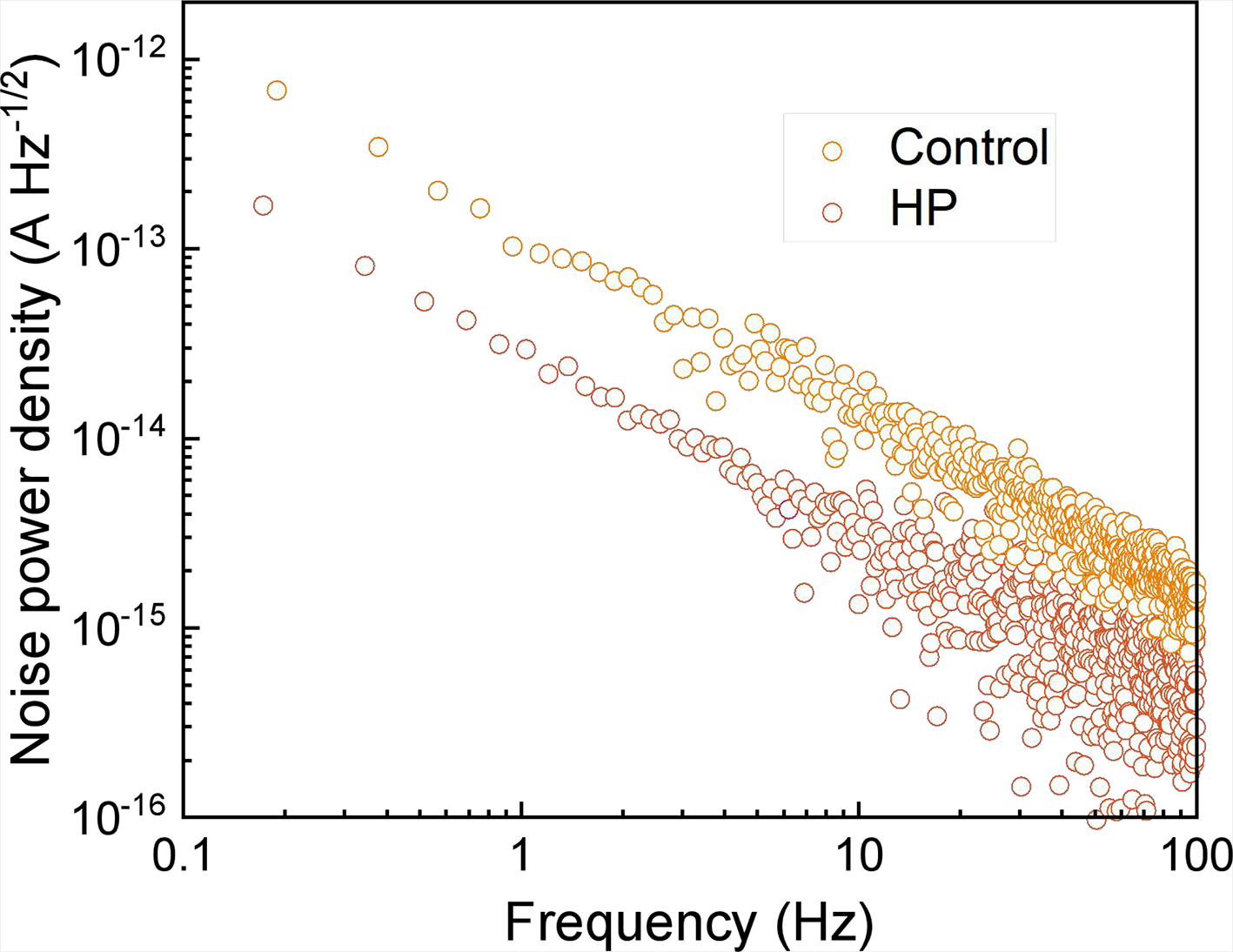


**Figure S8.** Noise current of the control device and HP device as a function of frequency.


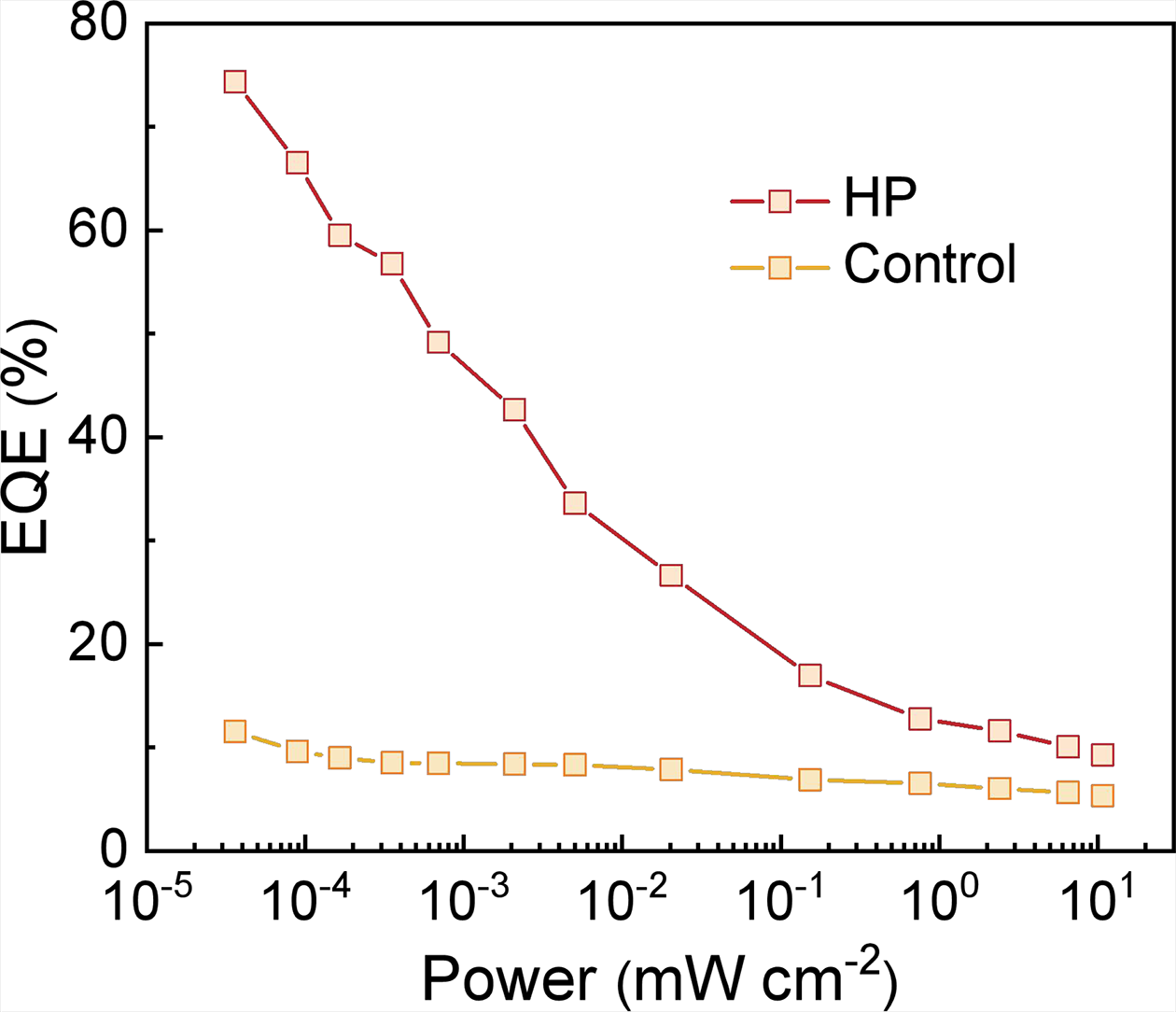


**Figure S9**. EQE versus light power curves of the control and HP devices at 0 V.

**
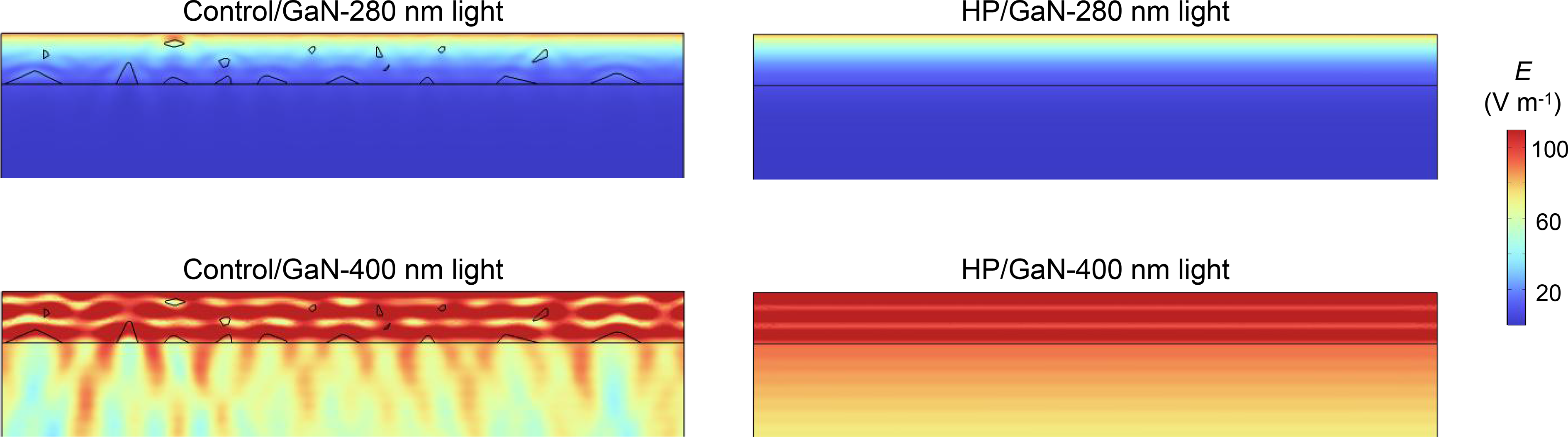
**

**Figure S10.** Calculated optical field distributions of the control device (left) and HP device (right) at 280 nm and 400 nm incident wavelengths, respectively. The thickness of the upper Cs_3_Cu_2_I_5_ layer is 300 nm.


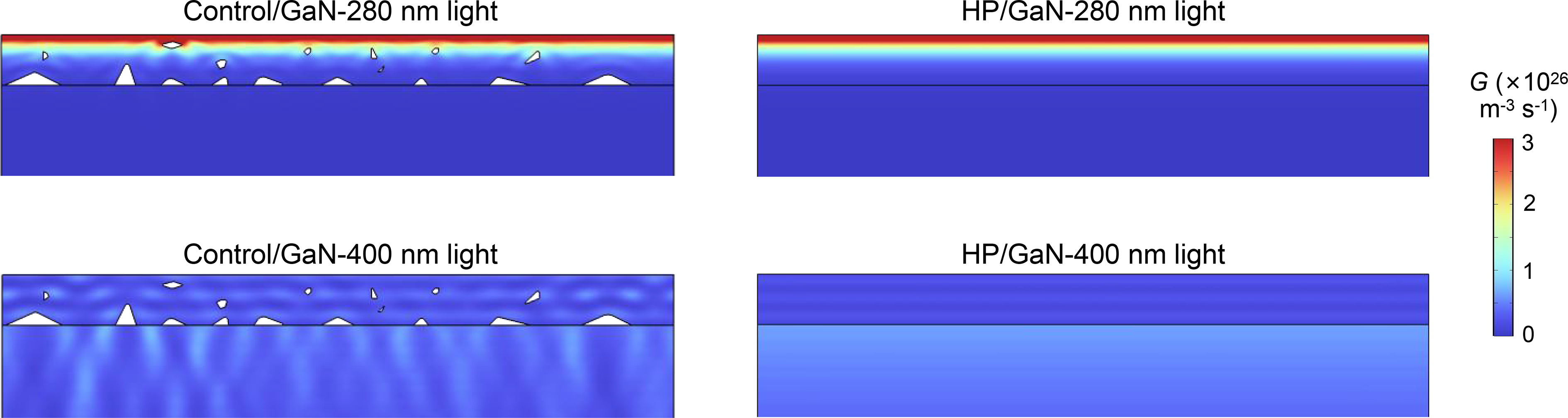


**Figure S11.** Calculated photogenerated carrier distributions of the control device (left) and HP device (right) at 280 nm and 400 nm incident wavelengths, respectively. The thickness of the upper Cs_3_Cu_2_I_5_ layer is 300 nm.


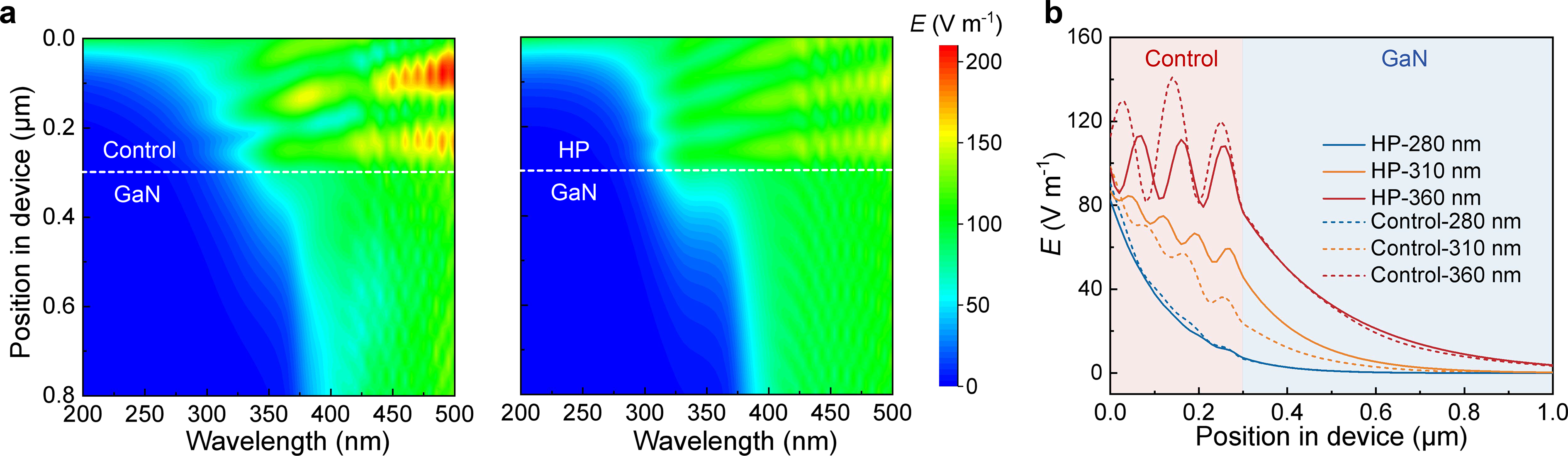


**Figure S12.** a) Simulated optical field distributions for the control device (left) and HP device (right) at incident wavelengths of 200–500 nm. b) Profiles of the photogenerated carrier distributions in the control device and HP device at 280 nm, 310 nm, and 360 nm incident light, respectively.


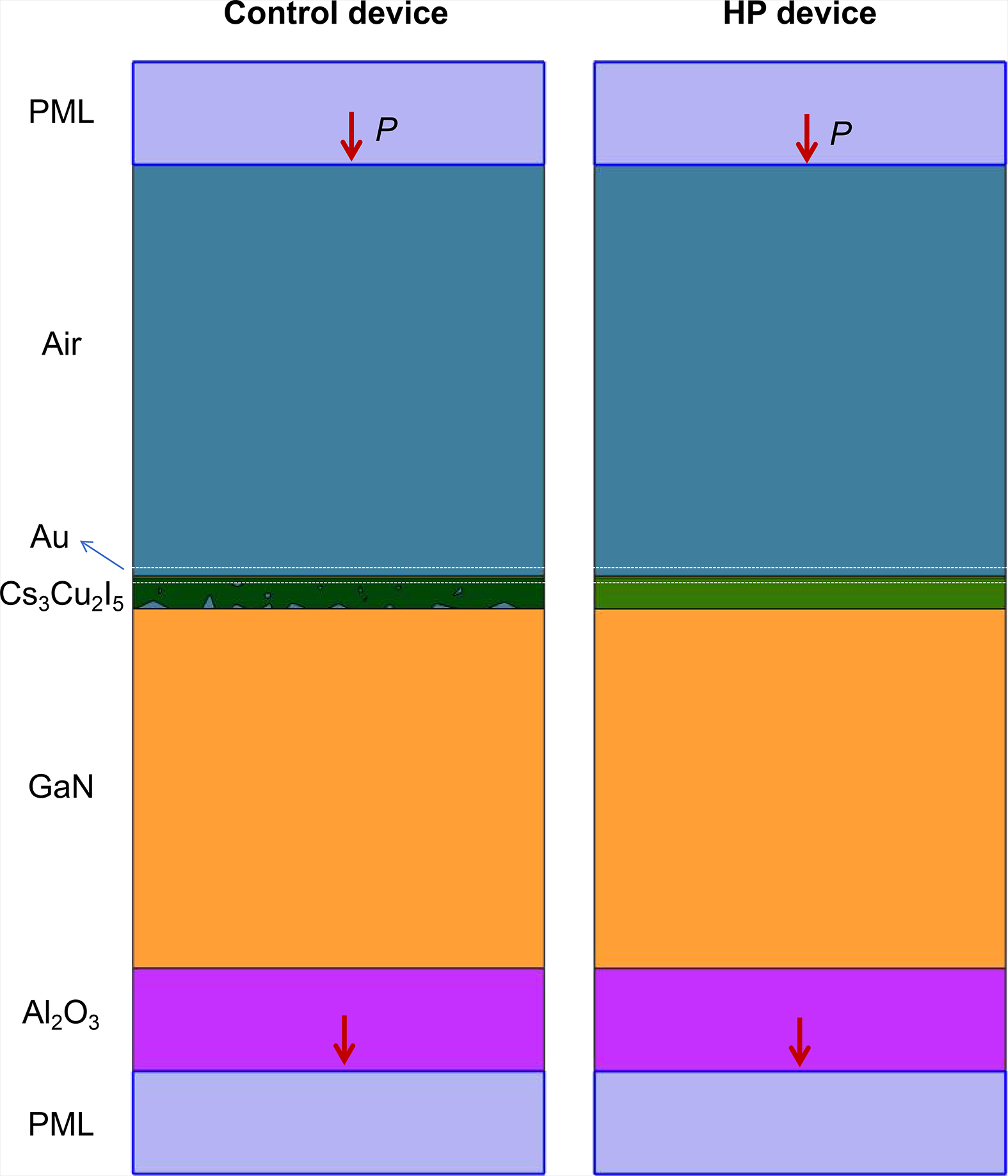


**Figure S13.** Two-dimensional geometric models of the control device and HP device established in the simulation.

**Table S1.** Multiplicity and total facet area percentage of (101), (110), (111), and (200) crystal face.

| Crystal face | Multiplicity | Total facet area percentage (%) |
| --- | --- | --- |
| (101) | 4 | 48.26 |
| (110) | 4 | 38.02 |
| (111) | 8 | 9.19 |
| (200) | 2 | 4.52 |

**Table S2.** Fitting parameters of the PL decay curves for control films and HP films.

|  | Control | TP |
| --- | --- | --- |
| *A*_1_ | 0.09 | 0.06 |
| Proportion (%) | 10% | 6% |
| *τ*_1_ (μs) | 0.20 | 0.28 |
| *A*_2_ | 0.84 | 0.87 |
| Proportion (%) | 90% | 94% |
| *τ*_2_ (μs) | 1.00 | 1.11 |
| *τ*_ave_. (μs) | 0.98 | 1.10 |

**Table S3. Comparison of key performance metrics of the as-prepared photodetector with other reported self-powered UV photodetectors.**

| Device structure | *R*  (A W^−1^) | *D*^*^  (Jones) | EQE  (%) | *Τ*_r_  (μs) | *τ*_f_  (μs) | Ref. |
| --- | --- | --- | --- | --- | --- | --- |
| Cs_3_Cu_2_I_5_/GaN | 0.22 | 6.40 × 10^11^ | 74 | 26 | 116 | This work |
| Ga_2_O_3_/Cs_3_Cu_2_I_5_ | 2.33 × 10^−3^ | 2.4 × 10^8^ | − | 3.7 × 10^4^ | 4.5 × 10^4^ | [1] |
| CsPbCl_3_/PbS | 0.22 | 4.06 × 10^13^ | − | 1.92 | 4.55 | [2] |
| MoS_2_/GaN | 0.187 | 2.34 × 10^13^ | − | 46.4 | 114.1 | [3] |
| NiO/ZnO | 0.085 | 1.74 × 10^12^ | − | − | − | [4] |
| PdSe_2_/GaN | 0.25 | 7.9 × 10^12^ | 86 | 28.5 | 122.6 | [5] |
| MAPbI_3_/ZnO | 0.026 | 4 × 10^10^ | − | 53 | 63 | [6] |
| PdSe_2_/FA_0.85_Cs_0.15_PbI_3_ | 0.31 | 1 × 10^13^ | 58 | 3.5 | 4 | [7] |
| Au/TiO_2_/P3HT | 2.5 × 10^−4^ | 1.09 × 10^10^ | − | 7.2 × 10^5^ | 5 × 10^5^ | [8] |
| GaN/ZnO | 2.82 | 6.82 × 10^13^ | − | 6.9 × 10^3^ | 6.4 × 10^3^ | [9] |
| MoS_2_/*β*-Ga_2_O_3_ | 2.05 × 10^−3^ | 1.21 × 10^11^ | − | − | − | [10] |
| GaN/Sn:Ga_2_O_3_ | 3.05 | 1.69 × 10^13^ | − | − | 1.8 × 10^4^ | [11] |
| *α*-Ga_2_O_3_/ZnO | − | 9.66 × 10^12^ | − | <50 | − | [12] |
| AlN/AlGaN | 0.025 | 1.4 × 10^12^ | 43 | − | − | [13] |
| (FAPbI_3_)_0.97_(MAPbBr_3_)_0.03_/Spiro-OMeTAD | 0.053 | 4.65 × 10^11^ | 37 | 4.6 × 10^4^ | 4.7 × 10^4^ | [14] |
| Cs_3_Cu_2_I_5_/Si | 0.13 | 3.1 × 10^10^ | − | 92.5 | 189.2 | [15] |
| GaS/GaN | 6.26 × 10^−3^ | 8.29 × 10^9^ | − | 48 | 80 | [16] |
| ZnO-Ga_2_O_3_ | 9.7 × 10^−3^ | 6.29 × 10^12^ | − | 100 | 900 | [17] |
| MAPbI_3_/CuO | 0.126 | 3.97 × 10^12^ | − | < 2 × 10^5^ | < 2 × 10^5^ | [18] |
| Cs_2_AgBiCl_6_/SnO_2_ | 9.8 × 10^−3^ | 5 × 10^11^ | 3.3 | − | − | [19] |
| ZnO/CuCrO_2_ | 3.43 × 10^−3^ | 8.5 × 10^9^ | − | 32 | 35 | [20] |
| Commercial UV-enhanced Si | 0.1 | 5 × 10^13^ | − | 0.006 | 0 | [21] |

**Table S4.** Fitting parameters of the electrochemical impedance spectroscopy for the control devices and HP devices.

|  | Control | HP |
| --- | --- | --- |
| *R*_s_ (Ω) | 40.7 | 15.2 |
| *R*_rec_ (MΩ) | 51.6 | 150 |

**Table S5.** Fitting parameters of the PL decay curves for the control films/GaN and HP film/GaN heterojunctions.

|  | Control | HP |
| --- | --- | --- |
| *A*_1_ | 0.33 | 1.05 |
| Proportion (%) | 33% | 71% |
| *τ*_1_ (μs) | 0.15 | 0.16 |
| *A*_2_ | 0.69 | 0.43 |
| Proportion (%) | 67% | 30% |
| *τ*_2_ (μs) | 1.01 | 1.01 |
| *τ*_ave_. (μs) | 0.96 | 0.78 |

**References**

1. J. Ma, X. Xia, S. Yan, Y. Li, W. Liang, J. Yan, X. Chen, D. Wu, X. Li, Z. Shi, Stable and Self-Powered Solar-Blind Ultraviolet Photodetectors Based on a Cs_3_Cu_2_I_5_/*β*-Ga_2_O_3_ Heterojunction Prepared by Dual-Source Vapor Codeposition. ACS Appl. Mater. Interfaces **2021**, 13, 15409−15419.
2. X. Zhan, X. Zhang, Z. Liu, C. Chen, L. Kong, S. Jiang, S. Xi, G. Liao, X. Liu, Boosting the Performance of Self-Powered CsPbCl_3_-Based UV Photodetectors by a Sequential Vapor-Deposition Strategy and Heterojunction Engineering. ACS Appl. Mater. Interfaces **2021**, 13, 45744−45757.
3. R. Zhuo, Y. Wang, D. Wu, Z. Lou, Z. Shi, T. Xu, J. Xu, Y. Tian, X. Li, High-Performance Self-Powered Deep Ultraviolet Photodetector Based on MoS_2_/GaN p–n Heterojunction. J. Mater. Chem. C **2018**, 6, 299−303.
4. C. Wei, J. Xu, S. Shi, Y. Bu, R. Cao, J. Chen, J. Xiang, X. Zhang, L. Li, The Improved Photoresponse Properties of Self-Powered NiO/ZnO Heterojunction Arrays UV Photodetectors with Designed Tunable Fermi Level of ZnO. J. Colloid Interface Sci. **2020**, 577, 279−289.
5. D. Wu, M. Xu, L. Zeng, Z. Shi, Y. Tian, X. J. Li, C. X. Shan, J. Jie, In Situ Fabrication of PdSe_2_/GaN Schottky Junction for Polarization-Sensitive Ultraviolet Photodetection with High Dichroic Ratio. ACS Nano **2022, *16*, 5545**−**5555.**
6. Z. Wang, R. Yu, C. Pan, Z. Li, J. Yang, F. Yi, Z. L. Wang, Light-Induced Pyroelectric Effect as an Effective Approach for Ultrafast Ultraviolet Nanosensing. Nat. Commun. **2015**, 6, 8401*.*
7. L. H. Zeng, Q. M. Chen, Z. X. Zhang, D. Wu, H. Yuan, Y. Y. Li, W. Qarony, S. P. Lau, L. B. Luo, Y. H. Tsang, Multilayered PdSe_2_/Perovskite Schottky Junction for Fast, Self-Powered, Polarization-Sensitive, Broadband Photodetectors, and Image Sensor Application. Adv. Sci. **2019**, 6, 1901134.
8. L. Zheng, X. Deng, Y. Wang, J. Chen, X. Fang, L. Wang, X. Shi, H. Zheng, Self-Powered Flexible TiO_2_ Fibrous Photodetectors: Heterojunction with P3HT and Boosted Responsivity and Selectivity by Au Nanoparticles. Adv. Funct. Mater. **2020**, 30, 2001604.
9. Y. Peng, J. Lu, X. Wang, W. Ma, M. Que, Q. Chen, F. Li, X. Liu, W. Gao, C. Pan, Self-Powered High-Performance Flexible GaN/ZnO Heterostructure UV Photodetectors with Piezo-Phototronic Effect Enhanced Photoresponse. Nano Energy **2022**, 94, 106945*.*
10. R. Zhuo, D. Wu, Y. Wang, E. Wu, C. Jia, Z. Shi, T. Xu, Y. Tian, X. Li, A Self-Powered Solar-Blind Photodetector Based on a MoS_2_/*β-*Ga_2_O_3_ Heterojunction. J. Mater. Chem. C **2018**, 6, 10982−10986.
11. D. Guo, Y. Su, H. Shi, P. Li, N. Zhao, J. Ye, S. Wang, A. Liu, Z. Chen, C. Li, W. Tang, Self-Powered Ultraviolet Photodetector with Superhigh Photoresponsivity (3.05 A/W) Based on the GaN/Sn:Ga_2_O_3_ p–n Junction. ACS Nano **2018**, 12, 12827−12835.
12. X. Chen, Y. Xu, D. Zhou, S. Yang, F. F. Ren, H. Lu, K. Tang, S. Gu, R. Zhang, Y. Zheng, J. Ye, Solar-Blind Photodetector with High Avalanche Gains and Bias-Tunable Detecting Functionality Based on Metastable Phase *α*-Ga_2_O_3_/ZnO Isotype Heterostructures. ACS Appl. Mater. Interfaces **2017**, 9, 36997−37005.
13. V. Kuryatkov, A. Chandolu, B. Borisov, G. Kipshidze, K. Zhu, S. Nikishin, H. Temkin, M. Holtz, Solar-Blind Ultraviolet Photodetectors Based on Superlattices of AlN/AlGa(In)N. Appl. Phys. Lett. **2003**, 82, 1323−1325.
14. T. M. H. Nguyen, S. Kim, C. W. Bark, Solution-Processed and Self-Powered Photodetector in Vertical Architecture Using Mixed-Halide Perovskite for Highly Sensitive UVC Detection. J. Mater. Chem. A **2021**, 9, 1269−1276.
15. W. Liang, Z. Shi, Y. Li, J. Ma, S. Yin, X. Chen, D. Wu, Y. Tian, Y. Tian, Y. Zhang, X. Li, C. Shan, Strategy of All-Inorganic Cs_3_Cu_2_I_5_ /Si-Core/Shell Nanowire Heterojunction for Stable and Ultraviolet-Enhanced Broadband Photodetectors with Imaging Capability. ACS Appl. Mater. Interfaces **2020**, 12, 37363−37374.
16. Z. Lin, T. Lin, T. Lin, X. Tang, G. Chen, J. Xiao, H. Wang, W. Wang, G. Li, Ultrafast Response Self-Powered UV Photodetectors Based on GaS/GaN Heterojunctions. Appl. Phys. Lett. **2023**, 122, 131101.
17. B. Zhao, F. Wang, H. Chen, L. Zheng, L. Su, D. Zhao, X. Fang, An Ultrahigh Responsivity (9.7 mA W^−1^) Self-Powered Solar-Blind Photodetector Based on Individual ZnO–Ga_2_O_3_ Heterostructures. Adv. Funct. Mater. **2017**, 27, 1700264.
18. H. Sun, W. Tian, F. Cao, J. Xiong, L. Li, Ultrahigh-Performance Self-Powered Flexible Double-Twisted Fibrous Broadband Perovskite Photodetector. Adv. Mater. **2018**, 30, 1706986.
19. M. Wang, P. Zeng, Z. Wang, M. Liu, Vapor-Deposited Cs_2_AgBiCl_6_ Double Perovskite Films Toward Highly Selective and Stable Ultraviolet Photodetector. Adv. Sci. **2020**, 7, 1903662.
20. T. Cossuet, J. Resende, L. Rapenne, O. Chaix-Pluchery, C. Jiménez, G. Renou, A. J. Pearson, R. L. Z. Hoye, D. Blanc-Pelissier, N. D. Nguyen, E. Appert, D. Muñoz-Rojas, V. Consonni, J. L. Deschanvres, ZnO/CuCrO_2_ Core–Shell Nanowire Heterostructures for Self-Powered UV Photodetectors with Fast Response. Adv. Funct. Mater. **2018**, 28, 1803142.
21. UV Enhanced 100% QE Photodiodes, OSI Optoelectronics, <https://osioptoelectronics.com/products/photodetectors/uv-enhanced-100-qe>, (accessed: December 2024).
